# A genetic strategy to measure insulin signaling regulation and physiology in *Drosophila*

**DOI:** 10.1371/journal.pgen.1010619

**Published:** 2023-02-02

**Authors:** Deborah D. Tsao, Kathleen R. Chang, Lutz Kockel, Sangbin Park, Seung K. Kim

**Affiliations:** 1 Department of Developmental Biology, Stanford University School of Medicine, Stanford, California, United States of America; 2 Department of Medicine (Division of Endocrinology, Metabolism, Gerontology), Stanford University School of Medicine, Stanford, California, United States of America; 3 Department of Pediatrics (Division of Endocrinology), Stanford University School of Medicine, Stanford, California, United States of America; 4 Stanford Diabetes Research Center, Stanford University School of Medicine, Stanford, California, United States of America; Instituto Leloir, ARGENTINA

## Abstract

Insulin regulation is a hallmark of health, and impaired insulin signaling promotes metabolic diseases like diabetes mellitus. However, current assays for measuring insulin signaling in all animals remain semi-quantitative and lack the sensitivity, tissue-specificity or temporal resolution needed to quantify *in vivo* physiological signaling dynamics. Insulin signal transduction is remarkably conserved across metazoans, including insulin-dependent phosphorylation and regulation of Akt/Protein kinase B. Here, we generated transgenic fruit flies permitting tissue-specific expression of an immunoepitope-labelled Akt (AktHF). We developed enzyme-linked immunosorption assays (ELISA) to quantify picomolar levels of phosphorylated (pAktHF) and total AktHF in single flies, revealing dynamic tissue-specific physiological regulation of pAktHF in response to fasting and re-feeding, exogenous insulin, or targeted genetic suppression of established insulin signaling regulators. Genetic screening revealed *Pp1-87B* as an unrecognized regulator of Akt and insulin signaling. Tools and concepts here provide opportunities to discover tissue-specific regulators of *in vivo* insulin signaling responses.

## Author summary

Insulin is an essential hormone that controls metabolism in all animals by regulating energy use and growth of target tissues. Impaired insulin signaling (“resistance”) in humans underlies development of type 2 diabetes, a pandemic disease causing significant morbidity and mortality. The genetic risk for insulin resistance is complex, and studies of diabetes are limited by a lack of tools to measure insulin resistance in a sensitive, quantitative, and tissue-specific way. Here, we describe a new technique to measure the strength of insulin signaling in fruit flies. By combining fruit fly genetics with antibody-based assays, we can quantify phosphorylated Akt, an evolutionarily conserved target of insulin signaling, in specific tissues of the adult fly. We show this technique can detect changes in insulin signaling after fasting and refeeding, addition of exogenous insulin, or genetic disruption of the insulin signaling pathway. We used this method to discover a new regulator of insulin signaling, a phosphatase enzyme encoded by *Pp1-87B*. This exciting new tool should advance our ability to study and discover additional regulators of insulin signaling and resistance.

## Introduction

Insulin is a crucial regulator of metabolism in multicellular animals, including the fruit fly *Drosophila melanogaster* [1]. Altered insulin sensitivity–like insulin resistance in liver, adipose and other insulin target tissues–can lead to pancreatic β cell failure and promote the pathogenesis of type 2 diabetes mellitus (T2DM) in humans [2–4]. Studies in patients with obesity and/or T2DM convincingly demonstrate a genetic basis for the pathogenesis of declining insulin sensitivity [5]. However, investigations of T2DM genetics are challenged to link altered gene function to *in vivo* phenotypes, and identify tissue(s) where gene function is required. *Drosophila* provides unique opportunities to dissect insulin signaling and resistance by combining genetics with appropriate physiological assays, like quantification of insulin signaling in target tissues. Moreover, the evolutionary conservation of this pathway could lead to discovery of novel insulin signaling regulators in mammals and humans.

*Drosophila* research has made important contributions to elucidating molecular and genetic regulation of insulin signaling in larval and adult organs [6,7], but progress in this area has also been hampered by a reliance on qualitative or semi-quantitative assays [8–10]. For example, signaling through insulin receptor (IR) and IR substrates (IRS1/2) leads directly to phosphorylation of Akt/Protein kinase B (hereafter called Akt), but assessment of this conserved feature of insulin signaling is limited in *Drosophila* to immune-staining of phospho-Akt (pAkt) in preserved tissues, or western blotting [9,10]. Similar limitations hamper studies of insulin signaling in other experimental systems, including mammals. An ideal *in vivo* assay for pAkt or Akt would permit absolute quantification from specific tissues at picomolar levels, but to date there is no such assay for flies or other metazoans.

Here, we describe generation of unique fly strains expressing *Drosophila* Akt tagged with two immunoepitopes (Hemagglutinin and FLAG: hereafter, AktHF). We developed ELISA methods to quantify phospho-AktHF (pAktHF) and total AktHF. Directed expression of a transgene encoding AktHF facilitated tissue-specific measures of pAktHF and total AktHF from single flies. These measures provided unprecedented *in vivo* assessments of insulin signaling and regulation by genetic and physiological mechanisms, and led to identification of previously undetected regulators of insulin-dependent Akt signaling. Our study provides potent new tools for studying insulin signaling, physiology and mechanisms underlying development of insulin resistance, and diseases like diabetes mellitus.

## Results

### A novel ELISA to measure *in vivo* Akt phosphorylation

**Fig 1:**
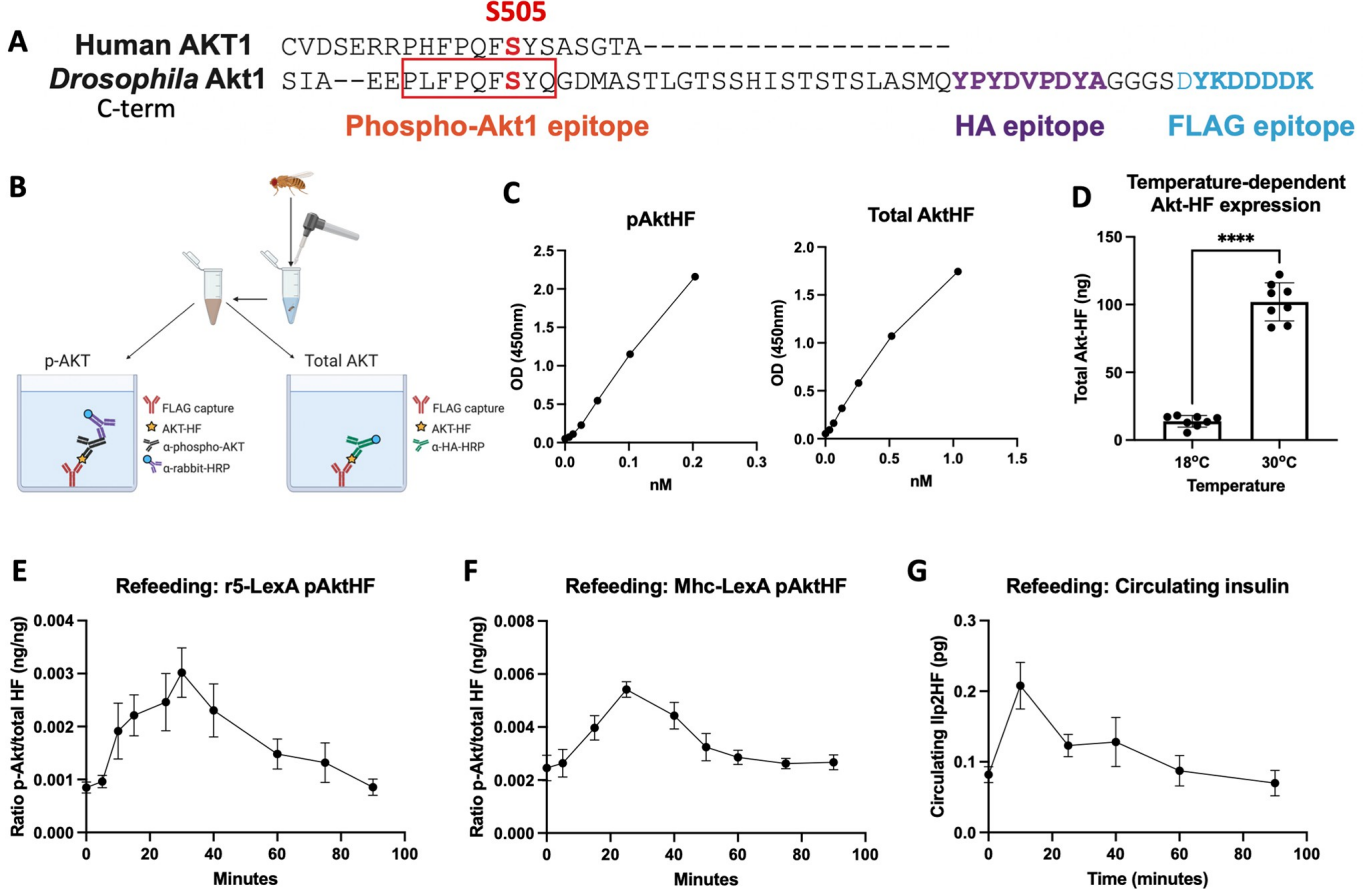
*Drosophila* pAktHF ELISA. **(A)** HA- and FLAG-tagged *Drosophila* Akt1 (AktHF), showing the conserved serine 505 phosphorylation site. **(B)** Schematic summary of the two parallel sandwich ELISAs for phosphorylated and total AktHF in a single adult fly. **(C)** Concentrations of phosphorylated and total AktHF were quantified by ELISA using phospho-Akt standard peptide (PLFPQFpSYQGDYKDDDDK, 0 to 0.5ng/mL, 0.2035nM) or FLAG(GS)HA standard peptide (DYKDDDDKGGGGSYPYDVPDYA, 0 to 2.5ng/mL, 1.036nM). **(D)** The expression of AktHF is under control of Gal80^TS^, shown at the inhibitory 18°C and the permissive 30°C (n = 8 flies per condition), two-tailed *t*-test *P*<0.0001. **(E)** Oral glucose-stimulated induction of AktHF phosphorylation in adult fat body (n = 4 flies per time point in one experiment). **(F)** Oral glucose-stimulated induction of AktHF phosphorylation in the adult muscle (n = 4 flies per time point in one experiment). **(G)** Oral glucose-stimulated insulin secretion and clearance in adult flies (n = 6 flies per sample, 3 samples per time point in one experiment).

To enable quantification of phospho-Akt (pAkt) in flies, we developed an enzyme-linked immunosorbent assay (ELISA) for an immuno-epitope tagged *Drosophila* Akt protein (**Fig 1A**) that can be expressed in any tissue/organ of interest using a tissue-specific binary expression system (either LexA/LexAop or Gal4/UAS; Methods). First, we generated and linked a transgene encoding Akt incorporating HemagglutininA (HA) and FLAG at the carboxy terminus (**Fig 1A**) to the LexA-responsive upstream activating sequence (*LexAop-AktHF*), and introduced a single copy of this construct into the *Drosophila* genome by site-directed insertion (Methods). Since general overexpression of Akt causes lethality in larvae, we used a temperature-sensitive Gal80 (*Gal80*^*TS*^) to restrict expression of AktHF to adult flies, and minimize or eliminate effects of Akt misexpression in developing tissues. In adult *Drosophila*, the fat body is an essential organ governing energy homeostasis that can store circulating fatty acids and glucose in response to insulin stimulation; thus, we focused our initial studies on the phosphorylation of Akt in this tissue using *r5*, a fat body-specific driver (**S1 Fig**) expressed from late embryo to adult stages [11]. To this end, we generated a triple-transgenic fly harboring *r5-LexA*, *Tubulin-Gal80*^*TS*^, and *LexAop-AktHF* where AktHF expression in adult fat body can be modulated by a temperature shift from a restrictive 18°C to a permissive 30°C. After rearing these flies at 18°C until eclosion–to suppress the expression of AktHF during development–we observed nearly normal developmental timing, with a time-to-eclosion delay of only 1 day compared to control single transgenic *LexAop-AktHF* flies or *r5-LexA* flies maintained at 18°C (**S2 Fig**). In subsequent experiments, AktHF expression was only induced at 30°C in the adult stage. After induction of AktHF at 30°C, we noted that total protein, triglyceride, and glycogen levels were indistinguishable from those in control single transgenic *LexAop-AktHF* flies (**S3A–S3C Fig**). Likewise, we observed similar survival following starvation after AktHF induction in bi-transgenic *r5-LexA*, *LexAop-AktHF* flies compared to single transgenic *r5-LexA* controls or *LexAop-AktHF* controls (**S3D Fig**), indicating that conditional AktHF production in adult flies did not alter systemic metabolism. Without *r5-LexA*, the *LexAop-AktHF* alone reporter was not expressed, even at the permissive temperature 30°C (**S4 Fig).**

To evaluate the expression level of AktHF in adult fat body, we homogenized individual triple-transgenic flies, then conducted two parallel ELISAs to measure total AktHF and phospho-AktHF (pAktHF) in lysates. For both ELISAs, we used an anti-FLAG capture antibody (Methods: **Fig 1**). Total AktHF was measured by ELISA using an anti-HA antibody, while pAktHF was measured using an anti-pAkt antibody recognizing phosphorylated serine 505 of *Drosophila* Akt (**Fig 1A and 1B**); this antibody also recognizes the orthologous phosphoserine 473 of Akt1 in mice and humans (Methods). To quantify the signal detected in these parallel assays, we synthesized two peptide ‘standards’: FLAG(GS)HA peptide for total AktHF quantification, and PLFPQFpSYSA(GS)FLAG peptide for phosphorylated AktHF quantification (Methods). Both total and pAktHF were measured in a linear range from 5–10 pM or higher (**Fig 1C**). Unphosphorylated Akt505 peptide standards were not detected in the phospho-Akt ELISA (Methods), demonstrating the specificity of this ELISA for phospho-AktHF and total AktHF quantification. We compared flies reared and maintained at 18°C, or reared at 18°C and maintained at 30°C for 48 hours, and observed induction of AktHF expression at 30°C (**Fig 1D**). The time course of AktHF expression and the detection of phosphorylated AktHF following this temperature shift is presented in **S4 Fig**. Thus, conditional expression of AktHF in adult fat body permitted quantification of total AktHF and pAktHF in a single fly at picomolar levels.

### Dynamic physiological regulation of Akt phosphorylation measured by ELISA

Pancreatic insulin secretion evoked by a meal stimulates Akt phosphorylation in mammalian target organs like liver, adipose and muscle [1]; thus, to assess the physiological relevance of our AktHF/pAktHF reporter system, we measured nutrient-dependent and insulin-dependent induction of pAktHF. Adult flies expressing *LexAop-AktHF* specifically in fat body were subjected to a fasting and glucose refeeding challenge. Compared to *ad libitum* fed flies, pAktHF levels were reduced after a fasting period (**S5 Fig**). After a 5-minute glucose re-feeding period followed by re-fasting, pAktHF levels rose for 30 minutes then declined thereafter to fasting levels by 90 minutes (**Fig 1E**). We observed similar dynamics in flies expressing *LexAop*-*AktHF* in muscle (**Fig 1F**), using the adult muscle-specific *Mhc-LexA* driver. Although the timecourse and magnitude of pAktHF changes were comparable between tissues, the ratio of phosphorylated-to-total AktHF was greater in muscle than fat body. In these studies, total AktHF did not significantly change during the period tested (**S6 Fig**), and we normalized pAktHF to total AktHF levels. Measures of circulating insulin levels in flies expressing Ilp2HF [12] showed that the excursion of circulating Ilp2HF (**Fig 1G**) corresponded well with tissue levels of pAktHF in this fasting-refeeding paradigm. Thus, we can detect dynamic, nutrient-dependent regulation of *in vivo* pAktHF levels in crucial organs regulating metabolism on a timescale of minutes. Using this fasting-refeeding paradigm, we also compared our pAktHF detection system to western blotting, the most widely used currently-available tool for measuring Akt phosphorylation in flies (**S7 Fig**). Compared to semi-quantitative measures of total and pAktHF afforded by western blotting (**S7A Fig**), ELISA using the same samples permitted pM quantification of total AktHF in a linear range using 1 to 3 flies (**S7B Fig**). After refeeding, ELISA also permitted pM quantification of pAktHF levels, also in a linear range using 1 to 3 flies (**S7C Fig**). Together, these findings supported use of 1 fly per sample in ELISA studies to measure total and pAktHF.

**Fig 2:**
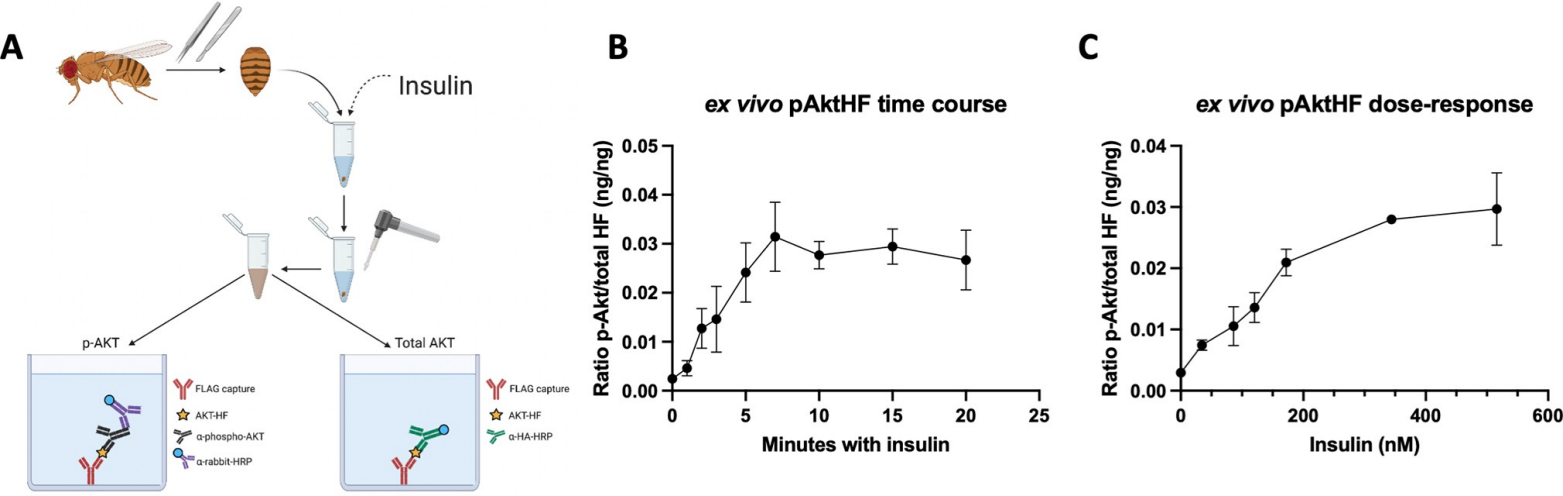
pAktHF *ex vivo* assay. **(A)** Schematic summary of the *ex vivo* AktHF ELISA, showing the dissection of the adult fat body and the addition of exogenous insulin. **(B)** Time course of insulin-stimulated phosphorylation of AktHF. (172nM human insulin, n = 4 fat bodies per time point in one experiment). **(C)** Dose-response curve for human insulin, assessed by the phosphorylation of AktHF (n = 4 fat bodies per concentration in one experiment).

To assess the dependence of AktHF phosphorylation on insulin signaling, we developed an analogous *ex vivo* pAktHF/AktHF ELISA assay based on addition of purified insulin (**Fig 2A**; Methods). After insulin addition to dissected abdominal fat body from individual *r5-LexA*, *LexAop-AktHF* flies, we observed rapid increase, then plateau of the pAktHF/total AktHF ratio within 10 minutes (**Fig 2B**). As expected, the amount of exogenous insulin determined the peak levels pAktHF in a dose-dependent manner (**Fig 2C)**. These findings provide strong evidence for insulin-dependent phosphorylation of AktHF and, together with our *in vivo* findings, support the view that the AktHF ELISA provides rapid, reproducible measures of insulin signaling and Akt phosphorylation in single adult flies, with unprecedented tissue specificity on a physiologically relevant timescale.

### Measuring dynamic changes of insulin signaling and sensitivity by pAktHF ELISA

**Fig 3:**
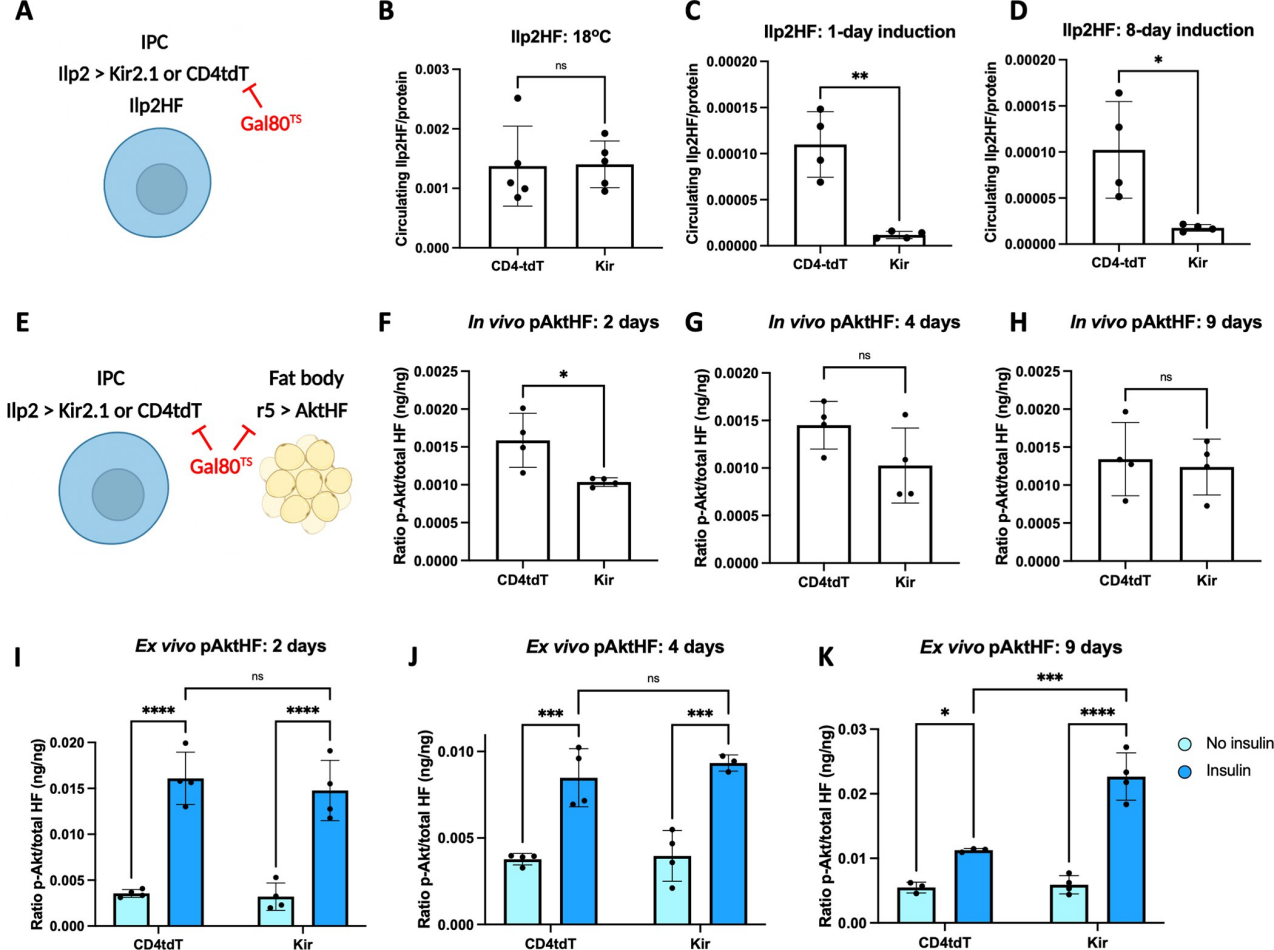
pAktHF ELISA can detect physiological changes of insulin sensitivity. **(A)** Genetics of Ilp2HF measurements (panels B-D), in which insulin-producing cells express either the inhibitory potassium channel Kir2.1 or the control CD4-tdTomato. IPCs also express a knock-in HA- and FLAG-tagged Ilp2 (Ilp2HF) used to measure circulating insulin. Transgene expression of Kir2.1 or CD4-tdTomato is under control of Gal80^TS^. **(B-D)** Measurements of circulating insulin at baseline (18°C, panel B), following a 1-day induction at 30°C (panel C), or following an 8-day induction at 30°C (panel D). *P* = 0.9346 (panel B), *P* = 0.0015 (panel C), *P* = 0.0181 (panel D). **(E)** Genetics of pAktHF measurements (panels F-H), in which IPCs express either Kir2.1 or CD4-tdTomato, while the fat body expresses AktHF. All transgene expression is under control of Gal80^TS^. **(F-H)** Measurements of *in vivo* pAktHF in the adult fat body following 2 (panel F), 4 (panel G), and 9 days (panel H) at 30°C, which permits the expression of Kir2.1 or CD4-tdTomato in IPCs. *P* = 0.0226 (panel F), *P* = 0.1190 (panel G), *P* = 0.7450 (panel H). **(I-K)** *Ex vivo* insulin-stimulated pAkt HF in the adult fat body following 2 (panel I), 4 (panel J), and 9 days (panel K) at 30°C. Human insulin was used at 172nM. The difference in insulin-stimulated pAkt between CD4tdT and Kir-expressing flies following 9 days of heat shock was statistically significant, *P* = 0.0009 (panel K). All panels show standard deviation. Data shown from two independent experiments. *P*-values were generated using a two-tailed *t*-test (panels B-D, F-H) or a two-way ANOVA (panels I-K). * indicates *P*<0.05, ** *P*<0.01, *** *P*<0.001 and **** *P*<0.0001. N.S. indicates statistically not significant.

Insulin signaling in target organs is altered by changes in insulin output, but these physiological signaling dynamics have not been previously measured in adult flies [1]. Here, we modulated insulin output in insulin producing cells (IPCs), then measured changes in pAktHF in the fat body. To simultaneously express transgenes in both IPCs and the fat body, we combined the Gal4/UAS and LexA/LexAop systems (**Fig 3A–3H**). With *Ilp2-Gal4* driving expression of *UAS-KCNJ2* in the IPCs and *r5-LexA* driving expression of *LexAop-AktHF* in the fat body, we could simultaneously silence IPC function and measure effects on fat body AktHF phosphorylation (**Fig 3E**). As previously reported [12], conditional expression of the inward-rectifying potassium channel KCNJ2 (Kir2.1) in IPCs (Methods, **Fig 3A**) led to durable reduction of circulating Ilp2HF (**Fig 3A–3D**). Within two days of KCNJ2 expression, we observed reduced *in vivo* pAktHF levels in fat body (**Fig 3F**). However, this effect waned over time and after 9 days of IPC silencing by KCNJ2, we found that *in vivo* pAktHF levels were indistinguishable from controls with normal insulin levels (**Fig 3G and 3H**).

Insulin sensitivity in target organs can adapt to changes of insulin output [1,3], and our findings suggested that insulin sensitivity in the fat body might be increased in adaptation to chronic hypoinsulinemia. To test this directly, we used the *ex vivo* pAktHF assay to measure fat body responses to insulin in flies with chronic hypoinsulinemia, or in control flies with normoinsulinemia (**Fig 3I–3K**). This revealed increased insulin sensitivity in fat body of flies with prolonged IPC silencing and insulin reduction, providing evidence of enhanced insulin sensitivity from chronic hypoinsulinemia. Thus, the combination of these distinct *in vivo* and *ex vivo* assays provides a powerful way to assess changes in both insulin signaling and sensitivity in metabolic organs over a physiologically relevant timescale.

### Assessing genetic insulin resistance by measuring AktHF phosphorylation

**Fig 4:**
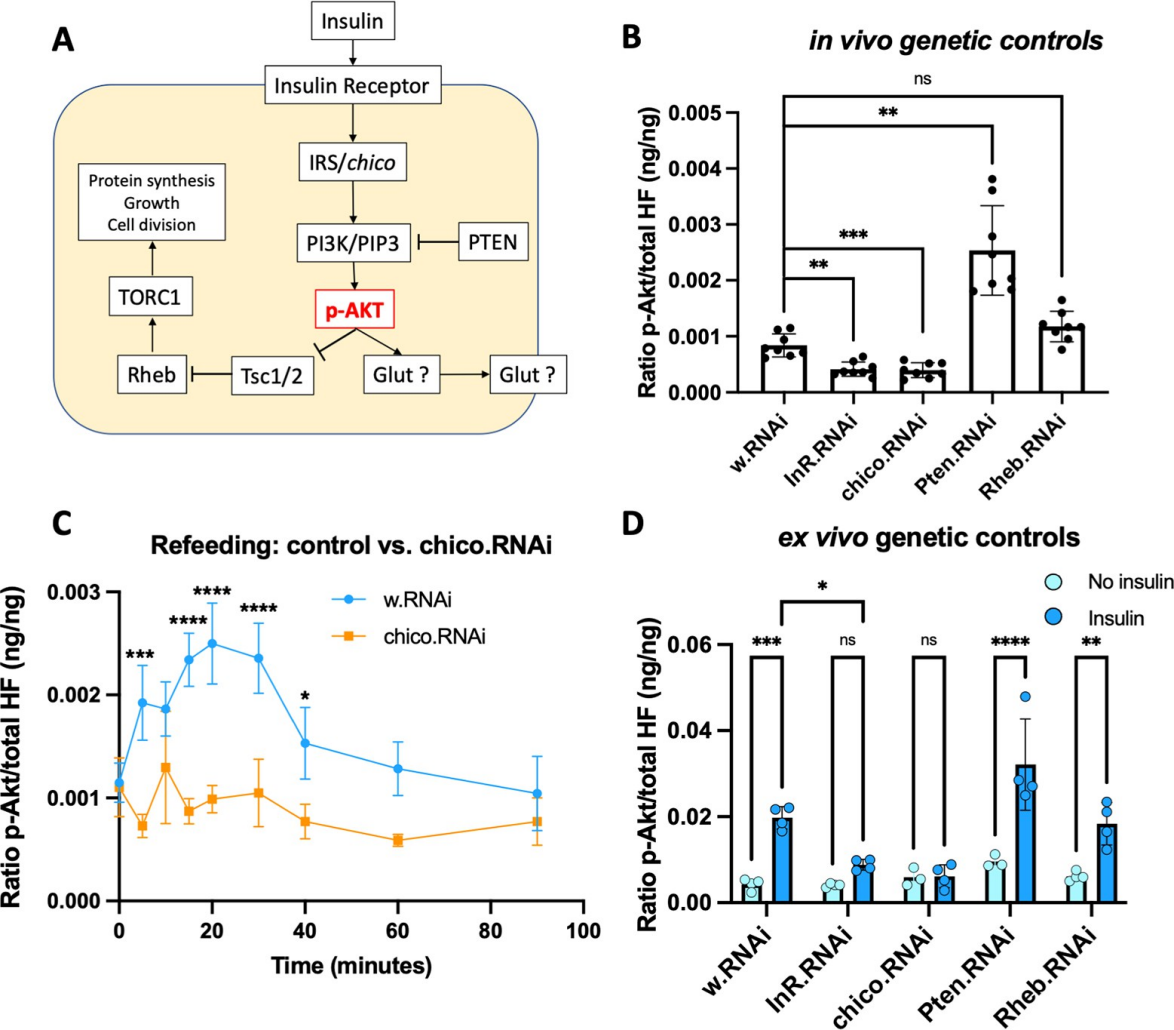
Genetic insulin resistance measured using the pAktHF ELISA. **(A)** Schematic of insulin signaling in metazoan cells. **(B)** *in vivo* measurement of pAkt following shRNA knockdown of various known insulin signaling components in the adult fat body (n = 8 flies per genotype in one experiment). Flies used had the genotype: *r5-LexA*, *Tub-Gal80*^*TS*^*/+*: *Ilp2-Gal4*, *LexAop-AktHF/LexAop-RNAi*. Compared to control *LexAop-w*.*RNAi*, *P* = 0.0017 (*LexAop-InR*.*RNAi*), *P =* 0.0001 (*LexAop-chico*.*RNAi*), *P =* 0.0018 (*LexAop-Pten*.*RNAi*), and *P =* 0.1178 (*LexAop-Rheb*.*RNAi*). **(C)** Oral glucose-stimulated AktHF phosphorylation in control *LexAop-w*.*RNAi* or *LexAop-chico*.RNAi flies (n = 4 flies per time point in one experiment). *P* = 0.0002 (5 min), *P*<0.0001 (15 min), *P*<0.0001 (20 min), *P* = 0.001307 (30 min). **(D)** Induction of pAktHF following *ex vivo* insulin stimulation in shRNA knockdown of known insulin signaling components. Human insulin was used at 172nM (n = 4 fat bodies per data point in one experiment). We observed a significant rise in pAktHF following insulin stimulation in control *LexAop-w*.*RNAi* flies (*P* = 0.0005), *LexAop-Pten*.*RNAi* (*P*<0.0001), and *LexAop-Rheb*.*RNAi* (*P* = 0.0082). Insulin-stimulated rise in pAktHF was not significant in *LexAop-InR*.*RNAi* (*P =* 0.8219), or *LexAop-chico*.*RNAi* (*P*>0.9999). P-values were generated using a two-tailed *t*-test (panels B-C) or a two-way ANOVA (Panel D). * indicates *P*<0.05, ** *P*<0.01, *** *P*<0.001, and **** *P*<0.0001. N.S. indicates statistically not significant.

Our findings suggested the pAktHF ELISA could be used to identify novel genetic regulators of insulin resistance, an established risk for type 2 diabetes [5]. To investigate this possibility, we first assessed pAktHF changes in flies after genetic suppression of known insulin signaling regulators (**Fig 4A**; [13]). After shRNA knockdown in fat body of the insulin receptor (*InR*) or *chico*, the *Drosophila* ortholog of IRS1/2, we observed a significant reduction of pAktHF in *ad libitum* fed flies (**Fig 4B**). As expected, knockdown of *Pten*, a phosphatase inhibitor of insulin signaling, led to increased pAktHF levels, indicating enhanced insulin signaling. By contrast, knockdown of *Rheb*, which encodes a GTP-binding protein thought to function ‘downstream’ of Akt, did not produce significant changes of pAktHF (**Fig 4B**). Since pAktHF dynamics depend on nutrient and insulin (**Fig 1E**), we next assessed AktHF regulation during refeeding of flies with impaired insulin signaling. In flies with fat body loss of *chico* and challenged by fasting and 5-minute glucose refeeding followed by re-fasting and sample collection, we observed blunted AktHF phosphorylation compared to controls (**Fig 4C**); this result supports findings observed in the *ad libitum-*fed state (**Fig 4B**). We found similar changes using our *ex vivo* assay (**Fig 4D**). We also observed similar changes in pAktHF levels after knockdown of *InR*, *chico*, *Pten* or *Rheb* in muscle (**S8 Fig).** These findings provide evidence that the pAktHF ELISA can readily quantify altered insulin signaling and sensitivity resulting from tissue-specific genetic manipulations.

### Discovering novel regulators of insulin resistance

**Fig 5:**
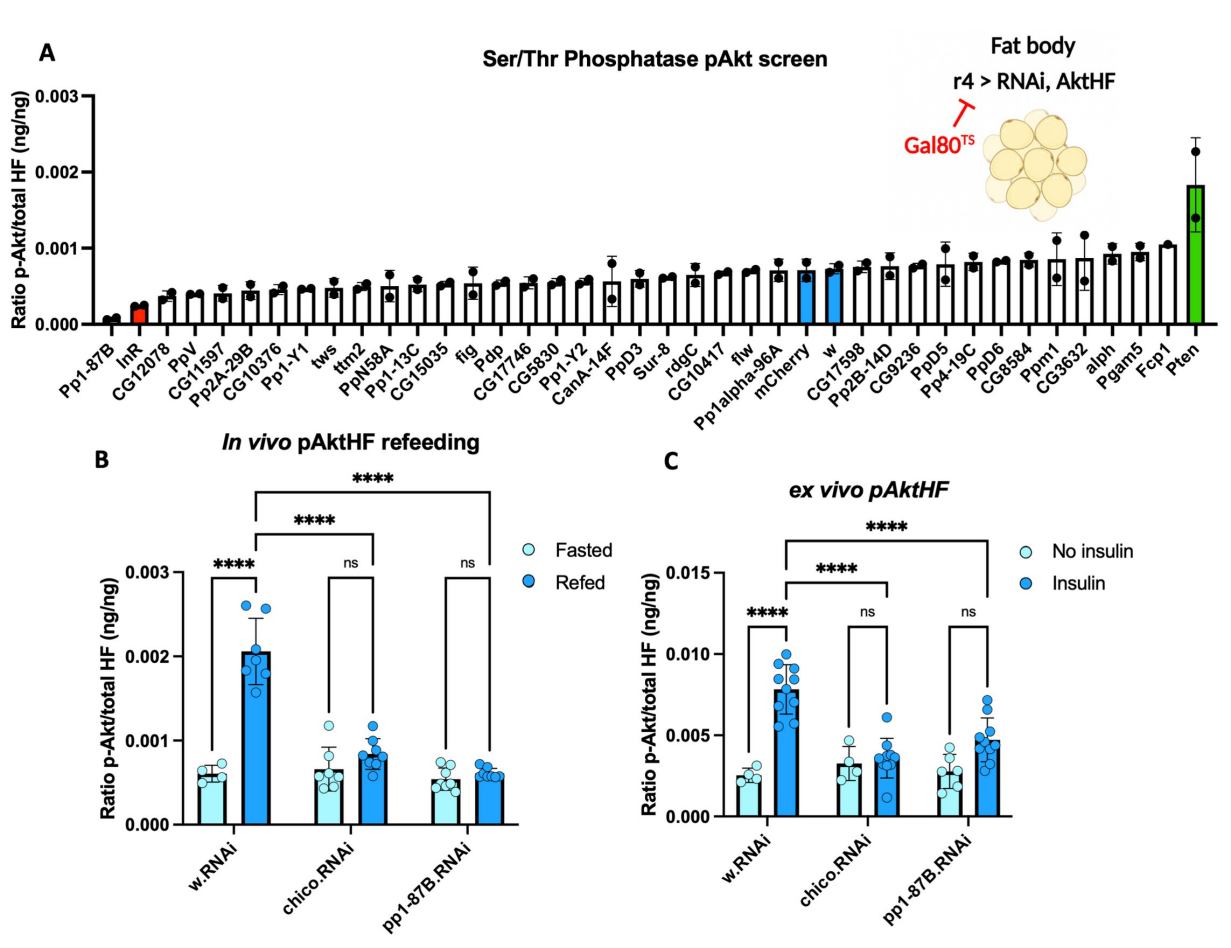
*In vivo* screen of Ser-Thr phosphatases for regulators of AktHF phosphorylation. **(A)** *In vivo* pAktHF screen of Ser/Thr phosphatase RNAi in the adult fat body. Includes negative controls (*UAS-mCherry*.*RNAi*, *UAS-w*.*RNAi*), and positive controls (*UAS-Pten*.*RNAi* and *UAS-InR*.*RNAi*) n = 2 flies per genotype. Representative results from two experiments shown. **(B)** Measurements of *in vivo* pAktHF in fasted flies or 20 minutes following an oral glucose challenge (two independent experiments). After re-feeding pAktHF was significantly lower in *UAS-Pp1-87B*.*RNAi* flies compared to control *UAS-w*.*RNAi flies* (*P*<0.0001). **(C)** Measurements of *ex vivo* pAktHF in dissected fat bodies before or after addition of human insulin (two independent experiments). The difference in insulin-stimulated pAkt was significantly lower in *Pp1-87B*.*RNAi* flies compared to control *w*.*RNAi* flies (*P*<0.0001). *P*-values were generated using a two-way ANOVA. ** indicates *P*<0.01, and **** *P*<0.0001. N.S. indicates statistically not significant.

Our assays provided an opportunity to identify novel regulators of insulin signaling, like *Drosophila* Serine/Threonine (Ser/Thr) phosphatases not previously associated with insulin signaling or Akt regulation. To facilitate efficient genetic screens using publicly available UAS-RNAi lines, we generated flies harboring *Lpp-Gal4*, a fat body specific driver, *UAS-AktHF*, and *Tubulin-Gal80*^*TS*^. The biological activity of *UAS-AktHF* expressed in the fat body using *Lpp-Gal4* was confirmed by measuring pAktHF dynamics during a fasting-refeeding challenge (**S9 Fig**), which showed that the rise and clearance of pAktHF resembled that observed using the *r5-LexA*, *LexAop-AktHF* system (**Fig 1E**). Likewise, UAS-shRNA-mediated knockdown of *InR* or *Pten* produced expected trending changes in pAktHF compared to *white* or *mCherry* controls (**Fig 5A**).

**Fig 6:**
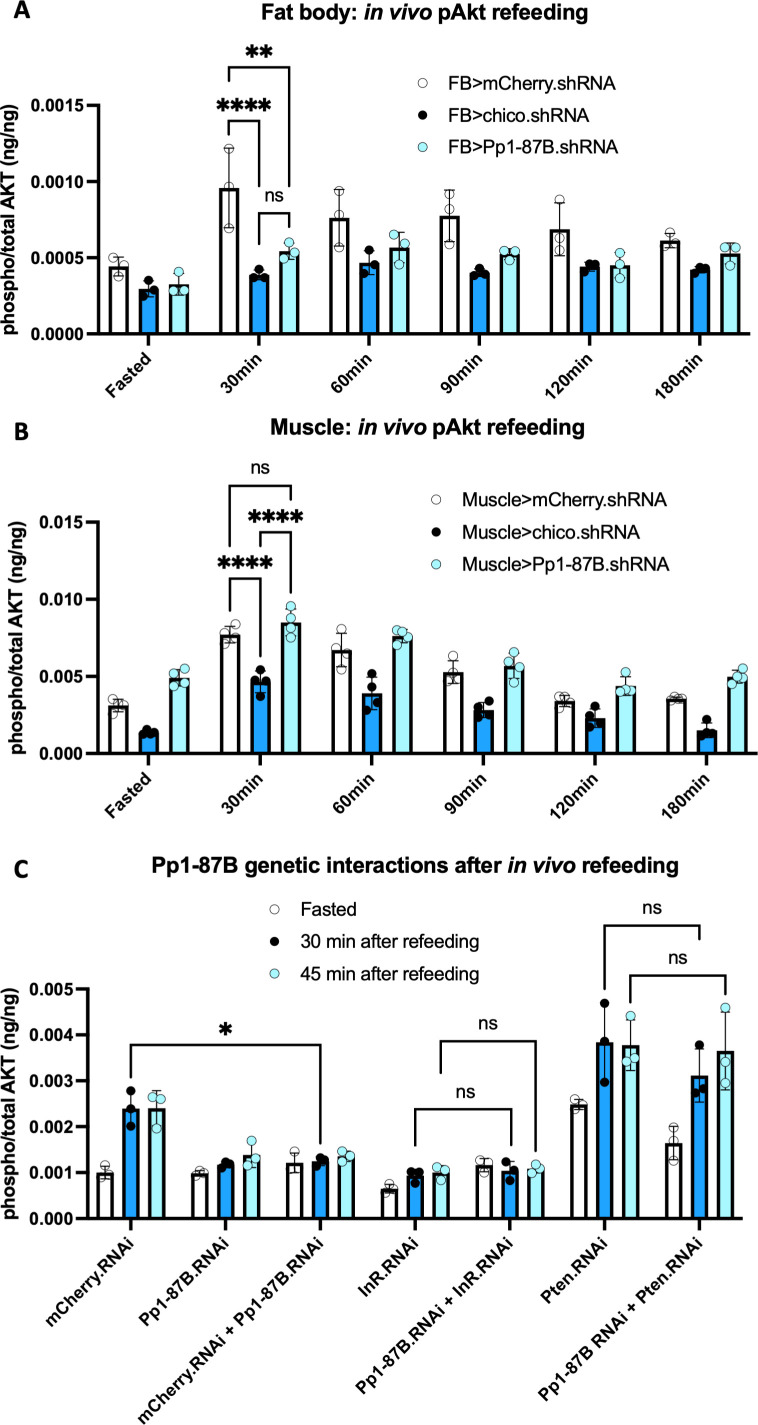
Tissue-specific roles and genetic interactions of Pp1-87B in insulin signaling regulation. **(A, B)** Measurements of *in vivo* pAktHF in the fat body of male flies with fat body-specific (A) or muscle-specific (B) knock down of *mCherry*, *chico*, or *Pp1-87B* genes in fasted and refed conditions. Measurements taken during fasting or 30, 60, 90, 120, or 180 minutes following an oral glucose challenge. In fat body after refeeding at 30 minutes, pAktHF was significantly lower in *Pp1-87B*.*RNAi* flies compared to *mCherry*.*RNAi* flies (P = 0.0102). In muscle after refeeding at 30 minutes, there was no significant difference in pAktHF levels between control and *Pp1-87B*.*RNAi* flies (P = 0.4096). **(C)** Genetic interaction of *Pp1-87B* and *Pten* for pAktHF measurements in adult male fat body at fasted and refed conditions. After refeeding, pAktHF was significantly lower in *mCherry*.*RNAi* + *Pp1-87B*.*RNAi* flies, compared to *mCherry*.*RNAi* alone (P = 0.0482). However, there was no significant difference in pAktHF between *InR*.*RNAi* versus *InR*.*RNAi* + *Pp1-87B*.*RNAi* (P>0.9999), and there was no significant difference between *Pten*.*RNAi* versus *Pten*.*RNAi* + *Pp1-87B*.*RNAi* (P = 0.6664).

We then measured the ratio of pAktHF/AktHF after shRNA-mediated knockdown of 35 Ser/Thr phosphatases in *ad libitum* fed flies (**Fig 5A**). After fat body-specific knockdown of *Pp1-87B*, which encodes the imputed *Drosophila* ortholog of the α and γ catalytic subunits of human protein phosphatase 1, we observed a trend towards reduced average levels of pAktHF (**Fig 5A)**. To validate this *in vivo* finding further, we performed a fasting-refeeding challenge. *Pp1-87B* fat body knockdown did not affect fat body pAktHF levels in fasted flies, but these flies had severely reduced pAktHF levels following glucose refeeding, a reduction comparable to that seen after knockdown of *chico* (females in **Fig 5B,** males in **S10 Fig)**. Flies of all genotypes in this experiment consumed a comparable amount of glucose during the refeeding period (**S11 Fig**). By contrast, flies with muscle-specific *Pp1-87B* knock-down had similar pAktHF levels relative to controls in a fasting-refeeding challenge (**Fig 6A and 6B**). Prior studies show that adult muscle expresses Pp1-87B at a comparable level to adult fat body [14]. Thus, our findings reveal that *Pp1-87B* regulation of AktHF phosphorylation may be tissue-specific.

Because feeding-dependent pAktHF dynamics could reflect regulation by signals other than insulin, we also measured the effect of *Pp1-87B* knock-down on insulin signaling using the *ex vivo* pAktHF assay. Using a fixed dose of insulin, fat bodies lacking Pp1-87B showed significantly diminished responsiveness to insulin stimulation (**Fig 5C**), indicating Pp1-87B regulates insulin signaling and Akt phosphorylation. To investigate this possibility further, we assessed the genetic interaction of *Pp1-87B* with *Pten*, a negative regulator of insulin signaling. Co-expression of shRNAs targeting both *Pp1-87B* and *Pten* in the adult male fat body resulted in the same pAktHF level as *Pten* shRNA (**Fig 6C**), indicating Pp1-87B regulates insulin signaling upstream of Pten. By contrast, shRNA suppression of insulin receptor (InR) and *Pp1-87B* reduced pAktHF levels to the same degree as *Pp1-87B* targeting alone (**Fig 6C**). Together, these results provide index evidence that loss of the *Drosophila* Ser/Thr phosphatase Pp1-87B in the fat body led to impaired insulin signaling.

## Discussion

Work here provides both conceptual and technical advances for studies of insulin signaling. The ELISA assay to measure AktHF or pAktHF quantifies insulin-dependent *Drosophila* Akt phosphorylation on a physiological timescale in single flies. Combination of this assay with powerful binary genetic expression systems additionally permits tissue-specific measures of *in vivo* insulin signaling and Akt phosphorylation without dissection; this includes studies of communication between insulin producing cells and insulin target organs, quantification of insulin resistance, and genetic screens to discover regulators of insulin signaling. These findings reveal uses of *Drosophila* to investigate hormonal and physiological regulation relevant to human metabolic diseases like obesity and diabetes.

Here, we demonstrated the insulin dependence of AktHF phosphorylation measured by the pAktHF ELISA, using physiological, genetic and biochemical approaches. Evidence here includes: (1) alignment of the kinetics of circulating insulin and pAktHF excursions in a fasting-refeeding paradigm (**Fig 1E–1G)**, (2) demonstration of AktHF phosphorylation after addition of insulin *ex vivo* (**Fig 2**), and (3) altered pAktHF levels after genetic loss-of-function targeting insulin signal transduction components (**Fig 4**). To the extent that *in vivo* pAktHF measurements in specific tissues reflect endogenous insulin signaling dynamics, we foresee that the sensitivity of the pAktHF ELISA–which permits measurements in single flies–should facilitate studies that complement or even substitute for current ELISA measures of circulating fly insulin excursions, which requires pooling of hemolymph from multiple flies [12,15]. Demonstration here that suppression of insulin secretion from adult IPCs reliably correlated with reduced pAktHF levels in fat body (**Fig 3**) further supports this possibility. For consistency, we used virgin female flies in the experiments shown in this study except where specifically indicated otherwise. However, the pAktHF ELISA can also be used in male flies, which demonstrate very similar fasting-refeeding dynamics and responses to genetic perturbations to the insulin signaling pathway (**S10 Fig**). Here we used the temperature-inducible Gal80^TS^ system to restrict AktHF expression to adult flies. We observed that overexpression of AktHF in larval stages appeared to be toxic to developing flies, as vials containing these flies had much lower rates of pupation. In contrast, overexpression of AktHF in the adult had minimal impact on survival or physiology (**S3 Fig**). Use of adult flies also permitted *in vivo* fasting-refeeding experiments to assess glucose reponsiveness of IPCs. Further studies are needed to generate an AktHF-based ELISA for use in larval stages, an important area of metabolism research [1].

To measure Akt phosphorylation, we labeled *Drosophila* Akt with the immuno-epitopes HA and FLAG, permitting quantification of AktHF or pAktHF, a general experimental strategy we previously used to measure dynamic *in vivo* circulating levels and total levels of Insulin-like peptide 2 [12]. *Drosophila* Akt has multiple regulatory phosphorylation sites [16] and the Ser505 and Thr342 residues have been previously studied [17]. The functional relevance of the vertebrate Akt1-Ser473 residue (which corresponds to *Drosophila* Akt-Ser505) has also been well-studied [18]. Phosphorylation of AktHF-Ser505 was detected with a pSer-specific antibody, a key reagent for building our pAktHF ELISA. Targeting of established ‘upstream’ regulators, like *InR*, *chico* and *Pten*, validated use of this ELISA to detect and measure changes of insulin signaling strength in adult flies. We speculate that identification of additional pSer or pThr antibodies [19] could be adopted to modify our ELISA strategy with AktHF. If so, measures of AktHF phosphorylation at other residues could also show specific kinetics and regulation by insulin signaling or other pathways.

Prior work suggests that Akt-pSer505 may be regulated by Akt-*dependent* signaling through the Tsc1/Tsc2-TOR-S6K pathway [10], and we have detected pAktHF changes in muscle after shRNA-based suppression of *Rheb*, the GTP binding protein downstream of Akt in the Tsc1/2-TOR pathway (**Figs 4A and S8**). In mammals, Akt-dependent signaling governs phosphorylation of factors like AMPK, and AS160, which regulate glucose transporters including Glut4 [20]; we speculate that development of phospho-protein ELISAs for ERK, AMPK or 4-EBP–analogous to the pAktHF ELISA–could lead to development of additional powerful quantitative assays to measure insulin signaling output at additional signaling detection end points. In addition to phosphorylation, and based on principles identified here and in prior work [12], we speculate that genetic approaches could also generate additional ELISAs to measure other chemically-stable covalent protein modifications governed by signaling relays.

The pAktHF ELISA described here is distinct and complementary to prior assays developed to assess or infer insulin signaling in fruit flies. These include western blotting for pAkt [21–25], immunohistological staining of phospho-Akt (pAkt) in fixed dissected tissues [9,10], or imaging of cellular localization of a pleckstrin homology domain-green fluorescent protein [26] to infer insulin receptor-dependent regulation of phosphoinositol-3 kinase (PI3K). While providing valuable insights about insulin signaling regulation, these qualitative or semi-quantitative assays lacked the temporal resolution, tissue specificity, or sensitivity of the pAktHF ELISA. ELISA-based approaches have previously been used to great effect to study *Drosophila* insulin-like peptides [21,27,28]. In addition, we show how the pAktHF ELISA can be adapted to permit *ex vivo* exposure to exogenous insulin that uncouples fly insulin signaling from endogenous insulin output, allowing unprecedented evaluation of insulin sensitivity in specific target tissues (**Figs 1 and 2**). While our assay could, in principle, detect Akt activation by multiple inputs, such as muscle-derived Pvf1 [29], our present study specifically interrogated insulin signaling inputs. Through the exogenous addition of purified insulin, the responses to acute refeeding, and the genetic perturbation of insulin signaling pathways, we can isolate specific inputs to Akt activation.

Here we combined the use of *in vivo* and *ex vivo* pAktHF ELISA to investigate and quantify dynamic changes of insulin signaling sensitivity resulting from hypoinsulinemia, over a timescale of days. Prior studies have shown that organisms can compensate for insulin resistance by increasing insulin output, while primary hyperinsulinemia leads to insulin resistance in target tissues [30,31]. Co-incidence of hypoinsulinemia and insulin hypersensitivity has been noted in fasted flies, Ames dwarf mice, pups of undernourished rats, and offspring of T2DM patients [9,32–34]. Here, we showed that electrical silencing of insulin-producing cells led to hypoinsulinemia and–initially–to reduced fat body pAktHF levels, as expected. However, pAktHF levels later normalized in these flies, suggesting adaptive enhancement of insulin signaling, and we used *ex vivo* pAktHF ELISA to reveal development of fat body hypersensitivity to insulin (**Fig 3**). Together, both assays provide novel measures and evidence of dynamic responses in peripheral insulin target organs to insulinemia. Future studies could address whether chronic hyperinsulinemia can also be reconstituted: if so, this could provide additional opportunities to assess insulin signaling adaptation in target organs.

Studies here also successfully used the pAktHF ELISA to identify genetic regulators of insulin resistance, including established factors (like *InR*, *chico* and *Pten*) and the phosphatase encoded by *Pp1-87B*. We show that this assay is sensitive to genetic perturbations, whether in single *ad libitum*-fed flies, refeeding-challenged flies, or in response to exogenous insulin. We used the pAktHF ELISA to quantify the ratio of phosphorylated-to-total AktHF, making it well-suited for detecting the impact of post-translational regulators like kinases and phosphatases on insulin signaling. Compared to kinase regulators of insulin signaling [1,6] phosphatases remain relatively understudied. *Drosophila* genetic screens have revealed phosphatases governing wing disc development, circadian systems, and embryonic development [13,35–37]. In a genetic screen of Ser/Thr phosphatases, we found that Pp1-87B suppression potently inhibited AktHF phosphorylation. Moreover, this result was confirmed using our *ex vivo* assay, indicating a requirement for Pp1-87B in regulating of insulin-dependent AktHF phosphorylation. In contrast to fat body suppression of Pten, suppression of Pp1-87B led to reduced pAktHF levels, indicating distinct mechanisms might mediate regulation of Akt phosphorylation by phosphatases like Pten and Pp1-87B. This effect appeared to be tissue-specific, as muscle-specific knockdown of Pp1-87B had no effect on insulin signaling. Pp1-87B is the *Drosophila* ortholog of the α and γ catalytic subunits of human protein phosphatase 1 (PP1), which has been the target of multiple human drug trials (NCT03886662, NCT03027388, NCT01837667) based on its postulated roles in cardiac function, immunity and cancer [38]. PP1 contains a catalytic subunit and one or several additional regulatory subunits which confer tissue and substrate specificity [27,39]. PP1 is a known target of Akt signaling and regulates phosphorylation of glycogen synthase to activate glycogen synthesis [40]. Genetic studies have identified a human T2DM risk variant rs4841132 within the long non-coding RNA *LOC157273* on chr8p23.1, associated with altered expression of *PPP1R3B* which encodes a PP1 regulatory subunit; this suggests a link between PP1 and T2DM or other metabolic traits [28,41]. However, it remained unknown if PP1 regulates Akt phosphorylation or insulin signaling: genetic suppression of the PP1 regulatory subunit PPP1R3G had no effect on Akt phosphorylation in mouse liver [40]. Findings here motivate future studies of mechanistic links between PP1 and Akt phosphorylation in flies and mammals, and genetic screens to discover additional regulators of insulin signaling.

## Materials and methods

### Generation of transgenic lines

To generate p13xLexAop2-IVS-Akt-HF (or LexAop-AktHF), a gene fragment consists of Drosophila Akt protein coding sequence (SD10374) without the stop codon, HA epitope coding sequence, a GGGS flexible hinge coding sequence, and FLAG epitope coding sequence was synthesized (Integrated DNA technologies), and cloned to NotI and XbaI sites on pJFRC19-13xLexAop2-IVS-myr-GFP vector [42]. The construct was inserted to PBac{y^+^-attP-9A}VK00027 site on the third chromosome by integrase-mediated transformation.

To generate pJFRC-MUH-Akt-HF (or UAS-AktHF), the same above gene fragment was cloned to NotI and KpnI sites on pJFRC-MUH vector [42]. The construct was inserted to PBac{y^+^-attP-9A}VK00027 site on the third chromosome by integrase-mediated transformation.

To generate pR5-BPnlsLexA::GADflUw (or r5-LexA), r tetramer [43] was PCR amplified from the genomic DNA of r4-Gal4 line (Bloomington stock #33832) using the primers GCCCTTTCGTCTTCAAGAATTCCTAGTCTTAAAATAATCAGGCGTAGAGTCAGAG and CGGGCGAGCTCGGCCGGCAGTCGACTGATCAGATCTTCGTAGGCC, and the largest PCR fragment was cloned to EcoRI and NaeI site on pBPnlsLexA::GADflUw vector [42]. Upon sequencing the construct, we found five repeats of 80 bp *Yp1* r enhancer were cloned, and thus named the construct as ‘r5-LexA’. The construct was inserted to P{CaryP}attP40 site on the second chromosome by integrase-mediated transformation. r5-LexA expression was confirmed by crossing to 13xLexAop-tdTomato.nls (Bloomington stock #66680) with minimal expression in adult optic lobes and restricted expression in abdomen and head fat body tissues, similar to r4-Gal4.

To generate pMhc.F3-580-BPnlsLexA::GADflUw (or Mhc-LexA), the adult leg muscle specific Mhc.F3-580 enhancer [44] was PCR amplified from the genomic DNA of y^1^ w^1118^ control flies using the primers ATGTTACAATTGAACTTATACCAACTCATTGGCTTTACAAG and CAGAATCTCGAGAGTCTCCCCTCTTACAACGATGTC, and cloned to EcoRI and XhoI (NaeI site modified) sites on pBPnlsLexA::GADflUw vector [42]. The construct was inserted to P{CaryP}attP40 site on the second chromosome by integrase-mediated transformation.

To generate pDilp215-1-BPnlsLexA::GADflUw (or Ilp2-LexA), 541 bp Dilp215-1 enhancer [45] from pDilp215-1-H-Stinger [46] was cloned to EcoRI and XhoI(NaeI site modified) sites on pBPnlsLexA::GADflUw vector [42]. The construct was inserted to P{CaryP}attP40 site on the second chromosome by integrase-mediated transformation.

To generate pDilp215-1-BPGUw (or Ilp2-Gal4), 715 bp EcoRI–KpnI fragment containing 541 bp Dilp215-1 enhancer and 155 bp Drosophila synthetic core promoter from pDilp215-1-BPnlsLexA::GADflUw was cloned to EcoRI and KpnI site on pBPGUw vector [42]. The construct was inserted to P{CaryP}attP2 site on the third chromosome by integrase-mediated transformation.

To generate Ilp2^HF^ knock-in allele (or Ilp2HF) in Ilp2 locus, two 20 bp guide RNA sequences, AGGCGAACTCGCCAACGGCA and TTACGCATGGCGCGCTTGTG, were cloned to pCFD3 vector (Addgene #49410). The donor construct pattB-Ilp2HF-DsRed-SCL was generated by inserting PBac{ScarlessHD-DsRed}module (Addgene #64703) into the TTAA sequence within the C-chain region of pBDP2-gd2HF [10]. The donor and two gRNA constructs were co-injected to vas-Cas9 embryos (Bloomington stock #55821), and F1 progeny were screened for DsRed eye color to identify successful knock-in events. PBac{ScarlessHD-DsRed} module was removed by Tub-PBac transposase (Bloomington stock #8285). The resulting Ilp2HF allele was sequenced to verify the reversion of TTAA sequence within the C-chain region. We note that Ilp2HF allele does not contain the endogenous Ilp2 intron, and Ilp2HF transcript level is about six times higher than wild type Ilp2 expression overall in 1, 5, and 10 day old males determined by qPCR.

### Drosophila strains and husbandry

For LexA phospho-Akt experiments, two fly lines were generated on a *y*^*1*^ *w*^*1118*^ (*yw*) background, with the following genotypes: (1) *yw; r5-LexA*, *Tubp-Gal80*^*TS*^*; Ilp2-Gal4*, *LexAop-AktHF* and (2) *yw; Mhc F3-580-LexA; Ilp2-Gal4*, *LexAop-AktHF*. These flies were crossed to *LexAop-RNAi* lines [13]. For Gal4 phospho-Akt experiments, flies were generated on the *yw* background, with the following genotype: *yw; Ilp2-LexA*, *Tubp-Gal80*^*TS*^*; Lpp-Gal4*, *UAS-AktHF*. These flies were crossed to *UAS-RNAi* lines obtained from the Bloomington Drosophila Stock Center. For Ilp2HF refeeding experiments, *yw; +; Ilp2HF*, *Ilp2-Gal4* flies were used. We used FlyBase as a resource on different transgenic constructs, fly genes, and curated publications [47].

Virgin female flies were used for all experiments except where specifically indicated otherwise. All flies were raised on a standard molasses and yeast-containing food media. Standard Drosophila cornmeal/molasses/yeast food was prepared with the recipe: 1% (w/v) agar, 5% (w/v) cornmeal, 10% (w/v) molasses, and 2.5% (w/v) baker’s yeast.

### Fasting and refeeding experiments

Flies were fasted for 28–70 hours in vials containing 2% agar. Fasting times depended on the age and genotype of the fly but were generally fasted until the first flies began to die of starvation to ensure robust refeeding. Flies were refed for 5 minutes on 250mM glucose, 2% agar, 1% blue food colouring. Following refeeding, they were returned to fasting vials. To collect flies for the phospho-Akt refeeding time course, 4 flies were shaken from the vials onto a CO_2_ pad, such that the rest of the flies in the vial remained awake. To reduce handling and stress to the animals, flies were returned to two separate fasting vials which were shaken on alternate time points. Robust refeeding was verified by the presence of blue colour in the abdomen. Single flies were placed in individual 1.5mL Eppendorf tubes and flash frozen in a solution of dry ice and 90% ethanol, before being transferred to -80°C until the conclusion of the time course. To collect flies for the Ilp2HF refeeding time course, flies were separated into separate vials for each time point to avoid repeated anesthetization.

### Ex vivo insulin stimulation

Fat bodies were isolated from live flies anesthetized on CO_2_ by isolating the abdomen and removing the ovaries and digestive tract. Dissections were performed in a drop of PBS on a glass slide. The fat body was placed in a 1.5mL Eppendorf tube with 100μl PBS. To stimulate insulin signaling, human insulin (Sigma-Aldrich I9278) was added at 172nM, unless otherwise indicated, and the tube was mixed by flicking. Unless otherwise specified, fat bodies were incubated with or without insulin for 10 minutes at 22°C.

### p-Akt ELISA

We coated wells in Nunc-Immuno modules (Thermo Scientific 468667) with 100μl of anti-FLAG antibody (Sigma-Aldrich F1804) diluted in 0.2 M sodium carbonate/bicarbonate buffer (pH9.4) to a final concentration of 2.5 μg/ml, then incubated for 16 hours at 4°C. The plate was then blocked with 350 μl of PBS containing 4% bovine serum albumin (Fisher Scientific BP1600) for 5 minutes at 22°C. The plate was washed three times with PBS containing 0.1% Triton-X.

A single intact fly or a single dissected fat body was placed on ice in 100 μl PBS with 1% Triton-X-100, 100mM sodium fluoride, and 5mM sodium orthovanadate. A pestle and cordless motor were used to lyse the tissue. The samples were centrifuged at 21,000g for 30 seconds at 4°C, and the supernatant was used for the ELISA.

For phosphorylated Akt measurement, supernatant was mixed with 50μl of Superblock containing 0.1% Triton X-100 and Phospho-Akt (Ser473) (D9E) XP Rabbit mAb (Cell Signaling Technology #4060) at a 1:2000 dilution. For total Akt measurement, supernatant was mixed with 50μl of Superblock containing 0.1% Triton X-100 and anti-HA-Peroxidase 3F10 antibody (Roche 12013819001) at a 1:10,000 dilution. Sample volumes for specific experiments are tabulated in S1 Methods. An equal volume of phospho-Akt standard peptide (PLFPQFpSYQGDYKDDDDK, 0 to 0.5ng/mL) or FLAG(GS)HA standard peptide (DYKDDDDKGGGGSYPYDVPDYA, 0 to 2.5ng/mL) was also loaded onto separate wells. The wells were sealed with an adhesive film (Bio-Rad MSB1001) and incubated on a rotary shaker at 4°C overnight.

Phospho-Akt wells were washed 6 times with PBS containing 0.1% Triton X-100. 100uL of PBS with 0.1% Triton X-100 and 4% BSA containing donkey anti-Rabbit-HRP (Thermo Fisher Scientific A16035) diluted 1:4000 was added to each well and incubated at room temperature on a rotary shaker for 1 hour. Phospho- and total-Akt wells were then all washed 6 times with PBS containing 0.1% Triton X-100. 100 μl of 1-Step Ultra TMB ELISA Substrate (Thermo Scientific 34029) was added to each well and incubated for 15 minutes at room temperature. The reaction was stopped by adding 50μl of 2M sulfuric acid, and the absorbance at 450 nm was immediately measured on a Varioskan LUX multimode microplate reader (Thermo Scientific VL0000D0).

### Ilp2HF ELISA

Circulating insulin was measured as previously described [12,15], with some small modifications. For flies homozygous for Ilp2HF, 6 flies were pooled in 57μl of extraction buffer (PBS with 0.1% Triton X-100). For flies heterozygous for Ilp2HF, 8 flies were pooled instead. Hemolymph was extracted on ice for 30 minutes. 42μl of extraction buffer was loaded for circulating Ilp2HF. Flies were then homogenized in 600uL (homozygous for Ilp2HF) or 800uL (heterozygous for Ilp2HF) extraction buffer, and 5μl supernatant was loaded for total Ilp2HF.

## Supporting information

S1 Fig Fat body-restricted expression pattern of *r5* (fat body) in adult *Drosophila*, visualized using *r5-LexA; LexAop-tdTomato*. (TIFF)

S2 Fig Comparison of time-to-eclosion for driver-only (*r5-LexA/+*, n = 13), reporter-only (*LexAop-AktHF/+*, n = 53), and double transgenic (*r5-LexA*, *Tubp-Gal80*^*TS*^*/+; Ilp2-Gal4*, *LexAop-Akt-HF/+*, n = 22) flies. All development occurred at 18°C, which suppresses the expression of AktHF.
(TIFF)

S3 Fig The expression of AktHF does not significantly alter the global metabolic profile of the fly. **(A-C)** Comparison of total-body protein (A), triglycerides (B), and glycogen (C) levels in adult *r5-LexA/+* flies or in *r5-LexA*, *Tubp-Gal80*^*TS*^*/+; Ilp2-Gal4*, *LexAop-AktHF/+* adult flies after 4 days’ induction of AktHF expression at 30°C. (A) *P* = 0.3806, (B) *P* = 0.9409, (C) *P* = 0.4473. **(D)** Comparison of survival following starvation in adult flies harboring *r5-LexA/+* (n = 21), *LexAop-AktHF/+* (n = 71), or both *r5-LexA* and *LexAop-AktHF* (n = 33). P-values were generated using a two-tailed *t*-test. N.S. indicates statistically not significant.
(TIFF)

S4 Fig The expression and phosphorylation of AktHF are induced following a temperature shift from 18°C to 30°C in flies harboring both *r5-LexA* and *LexAop-AktHF*. **(A)** Expression of total AktHF was induced after just one day at 30°C (*P*<0.0001), and increased after 5 days at 30°C (*P* = 0.0001). Without the *r5-LexA* driver, *LexAop-AktHF/+* flies did not express detectable AktHF at 30°C (*P*<0.0001). **(B)** Levels of phosphorylated AktHF were induced after just one day at 30°C (*P*<0.0001) and further increased after 5 days at 30°C (*P* = 0.003). Without the *r5-LexA* driver, *LexAop-AktHF/+* flies did not express detectable phosphorylated AktHF at 30°C (*P*<0.0001). *P*-values were generated using a two-way ANOVA. *** indicates *P*<0.001, **** indicates *P*<0.0001.
(TIFF)

S5 Fig AktHF phosphorylation decreases after a 24-hour fast (*P* = 0.001) and remains decreased after a 48-hour and 60-hour fast (*P* = 0.6585). (*P* = 0.9683 comparing 24- and 48-hour fast; *P* = 0.6585 comparing 48- and 60-hour fast). P-values were generated using a one-way ANOVA. *** indicates *P*<0.001, N.S. indicates statistically not significant.
(TIFF)

S6 Fig **(A, B)** Total levels of AktHF remain constant following refeeding challenge in fat body (A) or muscle (B). **(C)** Total levels of insulin (Ilp2HF) remain constant following refeeding challenge.
(TIFF)

S7 Fig **(A)** Western blots of 1–4 flies that had been fasted for 24 hours or fasted-then-refed with an oral glucose challenge. A single blot was sequentially probed for anti-HA-HRP, anti-pAkt.S505/anti-rabbit-HRP, and anti-Actin-HRP (arrow). **(B)** The quantification of pAktHF and total AktHF by ELISA assays. Two technical duplicates were assayed for each ELISA using the sample fly lysates for the western blots.
(TIFF)

S8 Fig *In vivo* measurement of pAktHF following shRNA knockdown of indicated known insulin signaling components in the adult muscle (n = 8 flies per genotype). Flies used had the following genetic background: *Mhc-LexA*, *Tubp-Gal80*^*TS*^*/+; Ilp2-Gal4*, *LexAop-AktHF/+*. Compared to the control *LexAop-w*.*RNAi*, *P* = 0.0380 (*LexAop-InR*.*RNAi*), *P =* 0.0002 (*LexAop-chico*.*RNAi*), *P =* 0.0003 (*LexAop-Pten*.*RNAi*), and *P* = 0.0150 (*LexAop-Rheb*.*RNAi*). P-values were generated using a two-tailed *t*-test * indicates *P*<0.05, and *** *P*<0.001. N.S. indicates statistically not significant.
(TIFF)

S9 Fig Oral glucose-stimulated induction of AktHF phosphorylation in adult fat body using the Gal4/UAS system. The genotype of virgin females used in these experiments is *Ilp2-LexA*, *Tubp-Gal80*^*TS*^*/+; Lpp-Gal4*, *UAS-AktHF/UAS-w*.*RNAi* (n = 4 flies per time point).
(TIFF)

S10 Fig Oral glucose-stimulated AktHF phosphorylation in control *LexAop-w*.*RNAi*, *LexAop-chico*.*RNAi*, or *LexAop-Pp1*.*87B*.*RNAi* male flies with a genetic background of *r4-LexA*, *Tubp-Gal80*^*TS*^*/+*: *Ilp2-Gal4*, *LexAop-AktHF/LexAop-RNAi* (n = 3 flies per time point in one experiment). (TIFF)

S11 Fig Flies of different genotypes immediately following a refeeding challenge with glucose containing blue dye. The ingested food can be visualized in the abdomen. Qualitatively, genotype differences did not affect the amount of food consumed.
(TIFF)

S1 Methods Supplementary methods are provided, including details on specific dilutions and loading volumes for ELISAs, quantification of protein, glucose, glycogen, and triglyceride content, the adult eclosion timing assay, the starvation survival assay, and western blotting. (DOCX)
