# Supplementary figures and images for: A genetic strategy to measure insulin signaling regulation and physiology in Drosophila
Source: PLoS Genet. 2023 Feb 2;19(2):e1010619. doi: 10.1371/journal.pgen.1010619 (PMC9928101; doi:10.1371/journal.pgen.1010619)

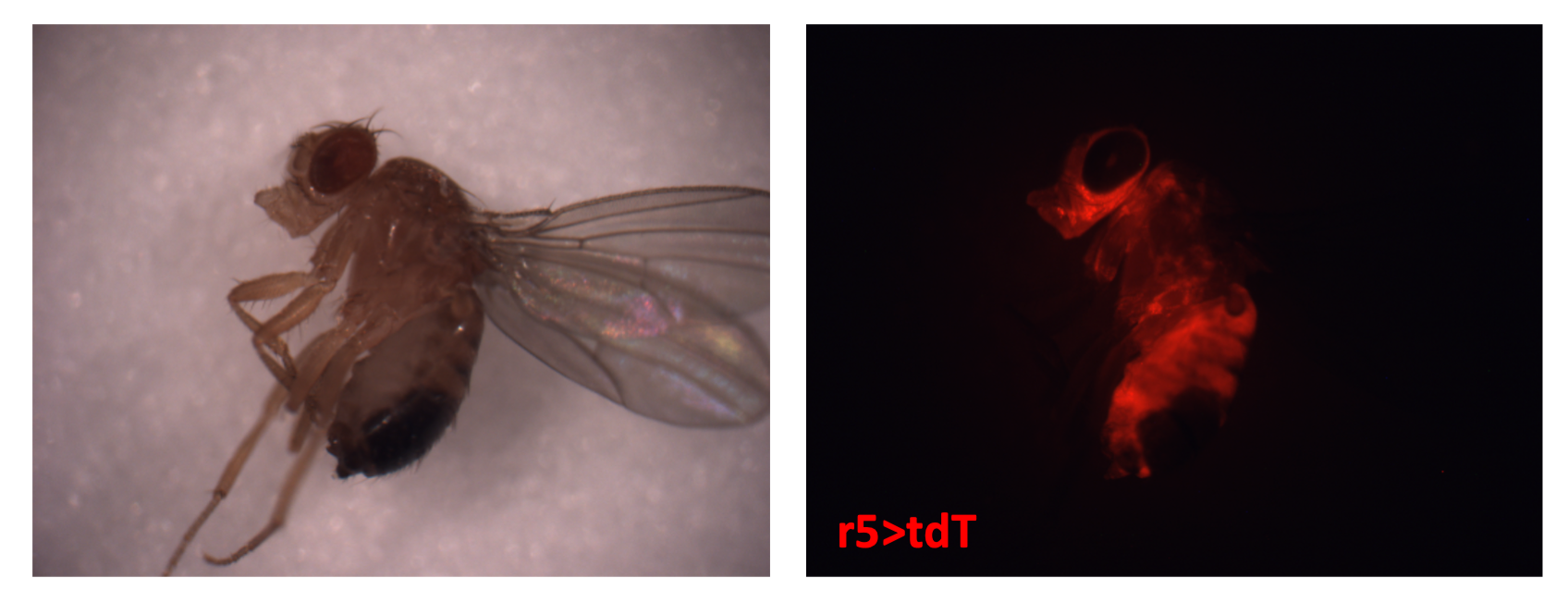

Supplement: S1 Fig — (TIFF) [file pgen.1010619.s001.tiff]

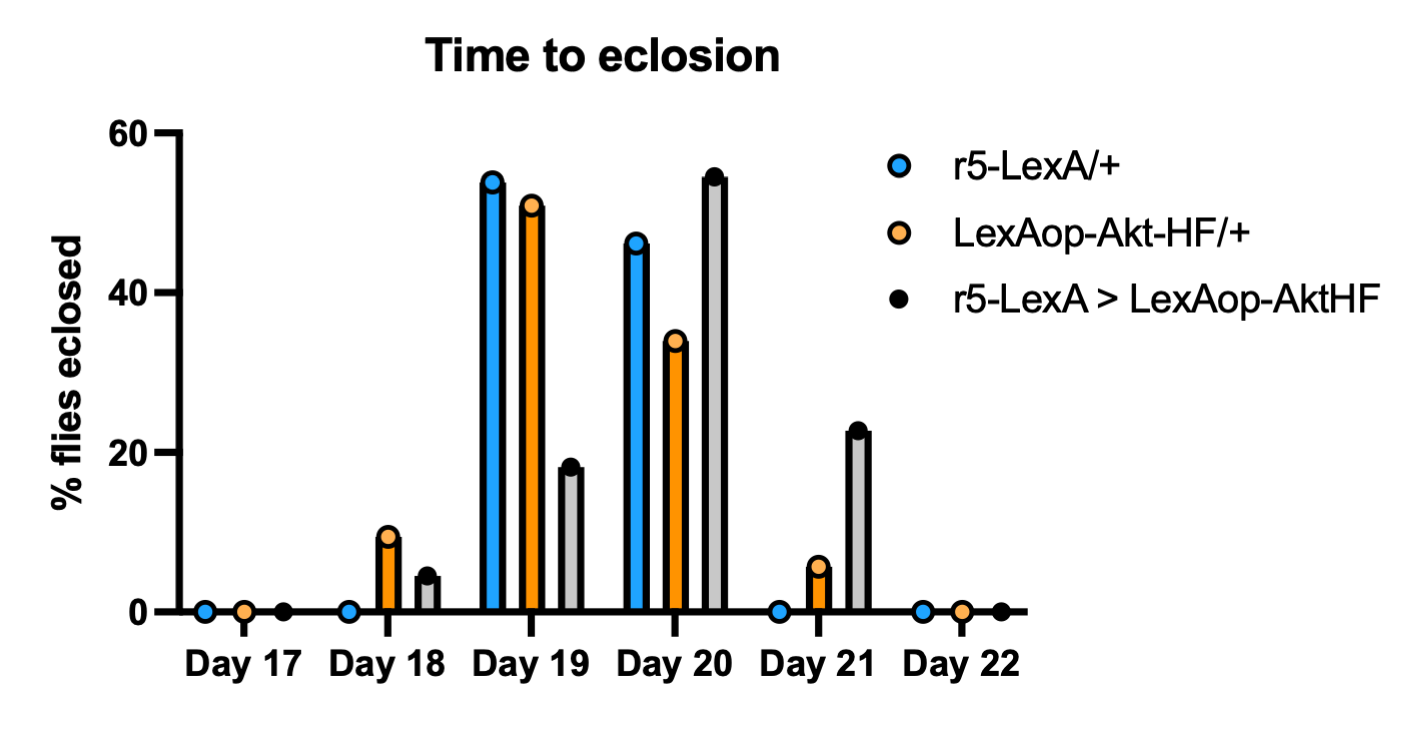

Supplement: S2 Fig — All development occurred at 18°C, which suppresses the expression of AktHF. (TIFF) [file pgen.1010619.s002.tiff]

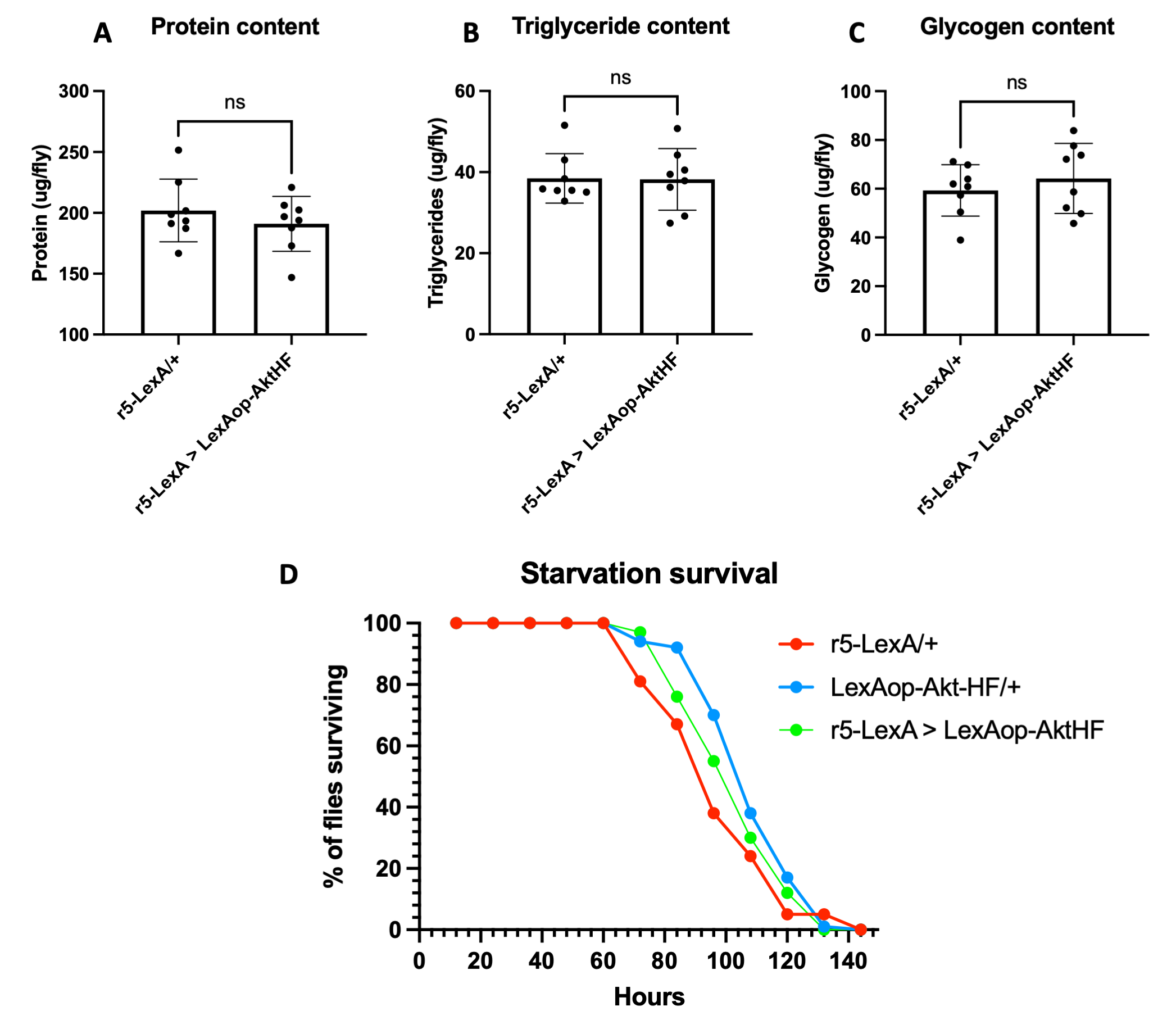

Supplement: S3 Fig — (A-C) Comparison of total-body protein (A), triglycerides (B), and glycogen (C) levels in adult r5-LexA/+ flies or in r5-LexA, Tubp-Gal80TS/+; Ilp2-Gal4, LexAop-AktHF/+ adult flies after 4 days’ induction of AktHF expression at 30°C. (A) P = 0.3806, (B) P = 0.9409, (C) P = 0.4473. (D) Comparison of survival following starvation in adult flies harboring r5-LexA/+ (n = 21), LexAop-AktHF/+ (n = 71), or both r5-LexA and LexAop-AktHF (n = 33). P-values were generated using a two-tailed t-test. N.S. indicates statistically not significant. (TIFF) [file pgen.1010619.s003.tiff]

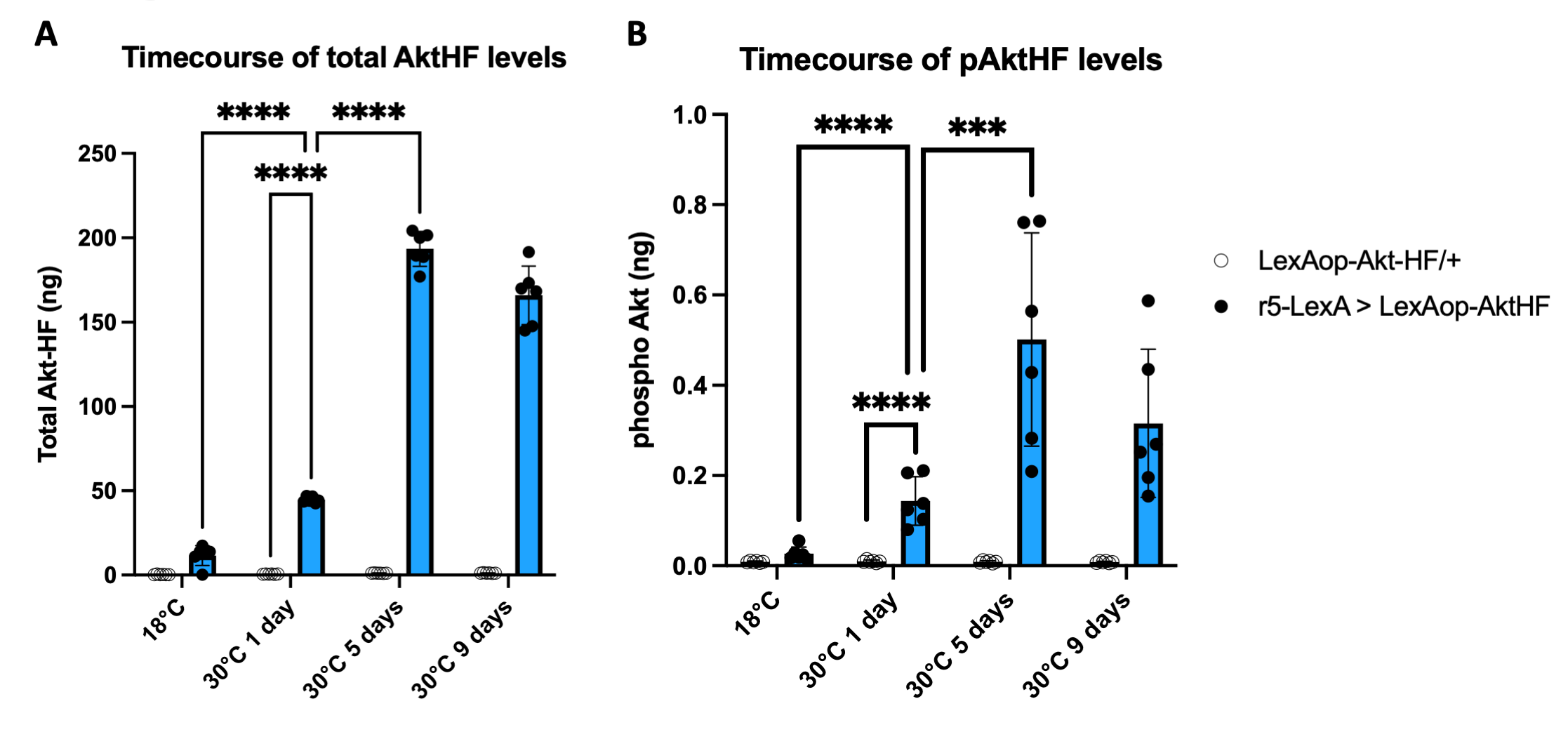

Supplement: S4 Fig — (A) Expression of total AktHF was induced after just one day at 30°C (P<0.0001), and increased after 5 days at 30°C (P = 0.0001). Without the r5-LexA driver, LexAop-AktHF/+ flies did not express detectable AktHF at 30°C (P<0.0001). (B) Levels of phosphorylated AktHF were induced after just one day at 30°C (P<0.0001) and further increased after 5 days at 30°C (P = 0.003). Without the r5-LexA driver, LexAop-AktHF/+ flies did not express detectable phosphorylated AktHF at 30°C (P<0.0001). P-values were generated using a two-way ANOVA. *** indicates P<0.001, **** indicates P<0.0001. (TIFF) [file pgen.1010619.s004.tiff]

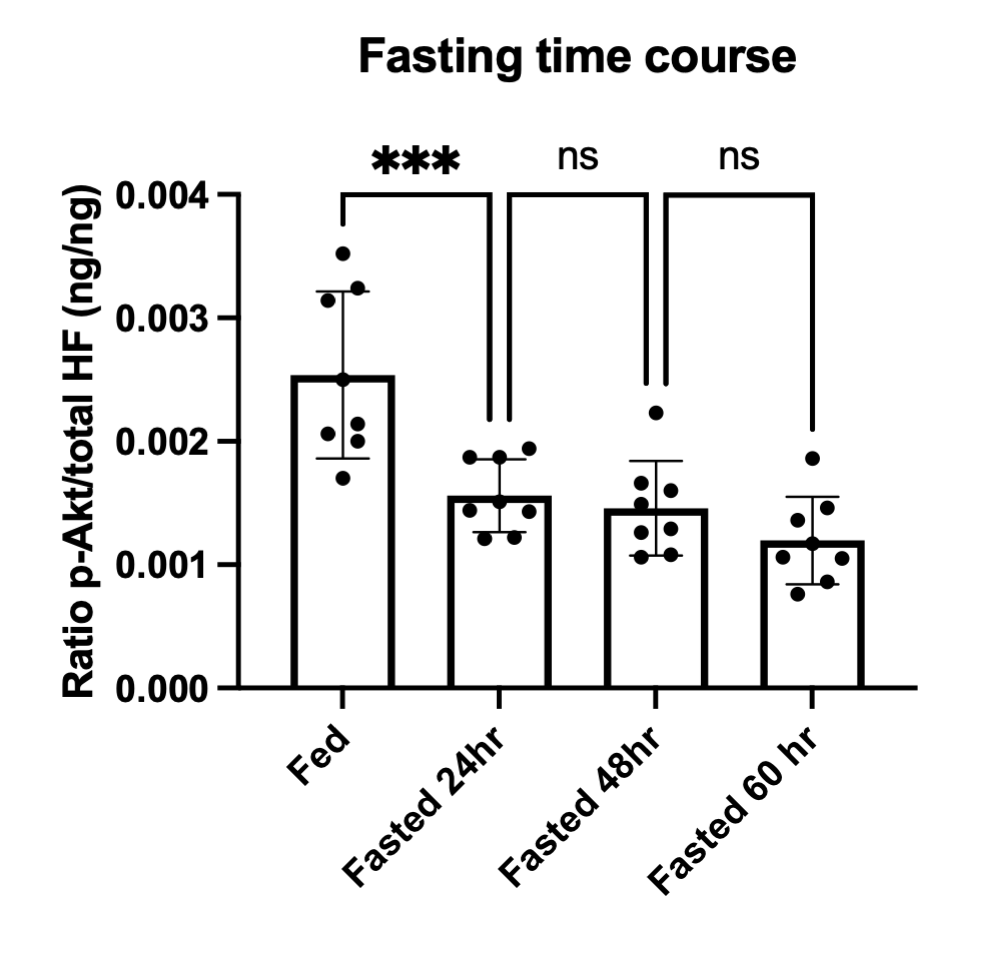

Supplement: S5 Fig — (P = 0.9683 comparing 24- and 48-hour fast; P = 0.6585 comparing 48- and 60-hour fast). P-values were generated using a one-way ANOVA. *** indicates P<0.001, N.S. indicates statistically not significant. (TIFF) [file pgen.1010619.s005.tiff]

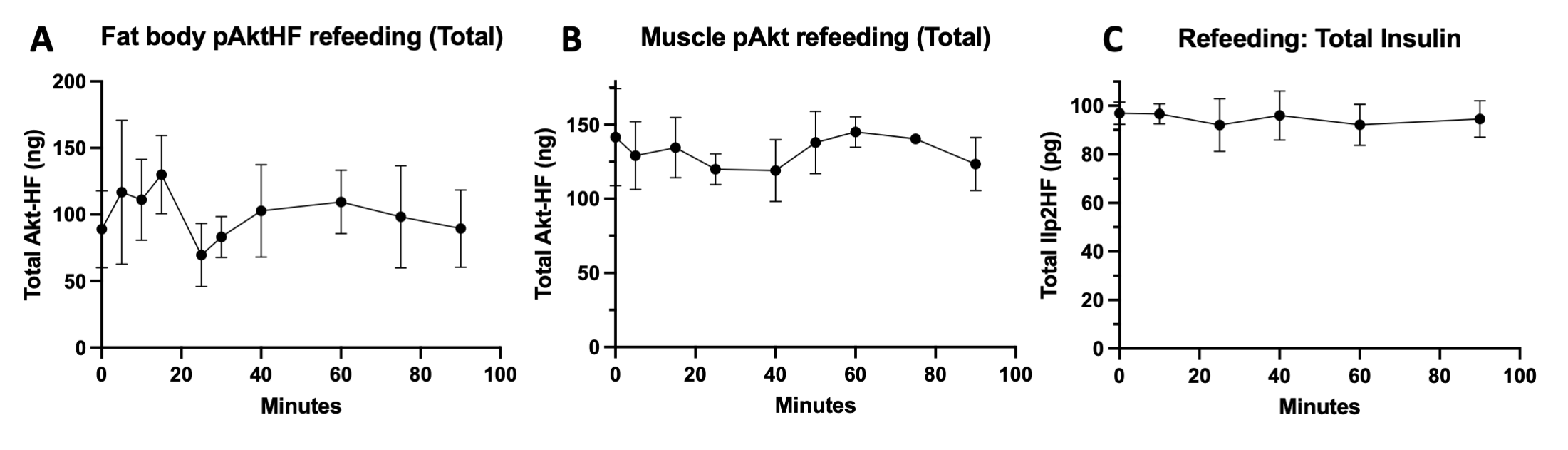

Supplement: S6 Fig — (A, B) Total levels of AktHF remain constant following refeeding challenge in fat body (A) or muscle (B). (C) Total levels of insulin (Ilp2HF) remain constant following refeeding challenge. (TIFF) [file pgen.1010619.s006.tiff]

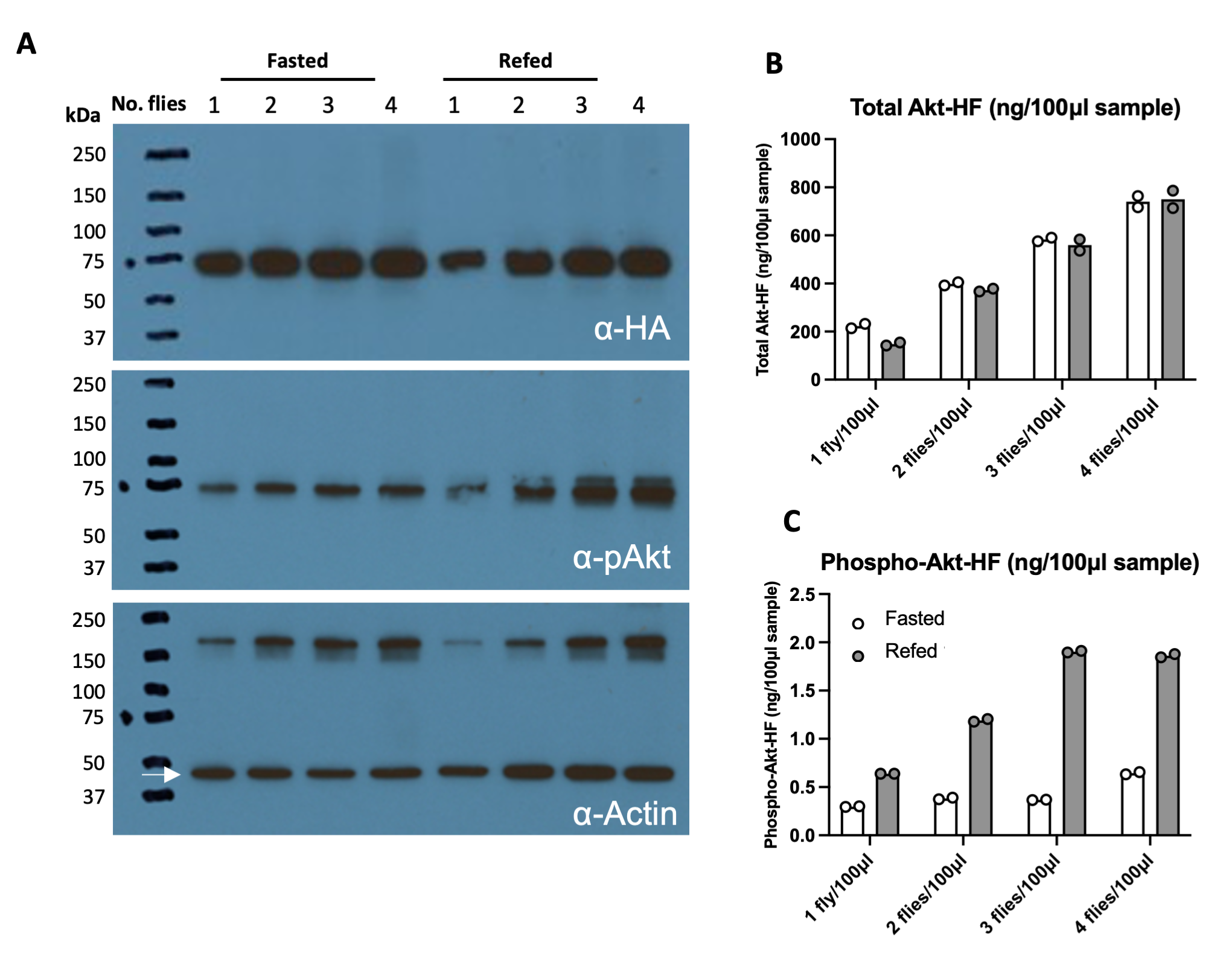

Supplement: S7 Fig — (A) Western blots of 1–4 flies that had been fasted for 24 hours or fasted-then-refed with an oral glucose challenge. A single blot was sequentially probed for anti-HA-HRP, anti-pAkt.S505/anti-rabbit-HRP, and anti-Actin-HRP (arrow). (B) The quantification of pAktHF and total AktHF by ELISA assays. Two technical duplicates were assayed for each ELISA using the sample fly lysates for the western blots. (TIFF) [file pgen.1010619.s007.tiff]

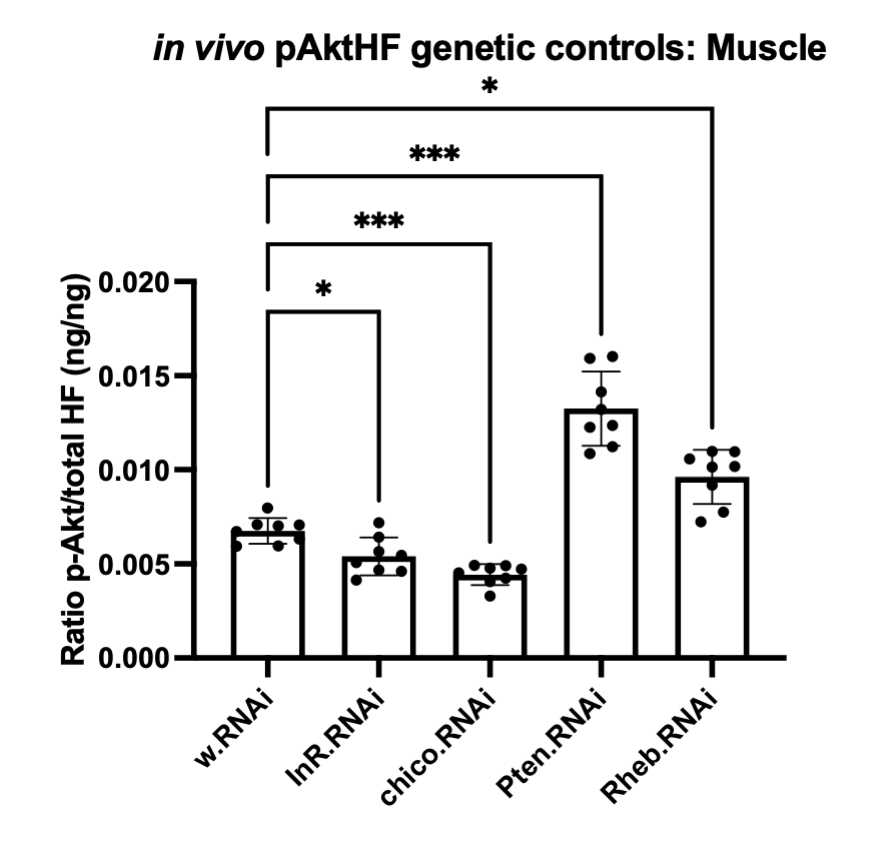

Supplement: S8 Fig — Flies used had the following genetic background: Mhc-LexA, Tubp-Gal80TS/+; Ilp2-Gal4, LexAop-AktHF/+. Compared to the control LexAop-w.RNAi, P = 0.0380 (LexAop-InR.RNAi), P = 0.0002 (LexAop-chico.RNAi), P = 0.0003 (LexAop-Pten.RNAi), and P = 0.0150 (LexAop-Rheb.RNAi). P-values were generated using a two-tailed t-test * indicates P<0.05, and *** P<0.001. N.S. indicates statistically not significant. (TIFF) [file pgen.1010619.s008.tiff]

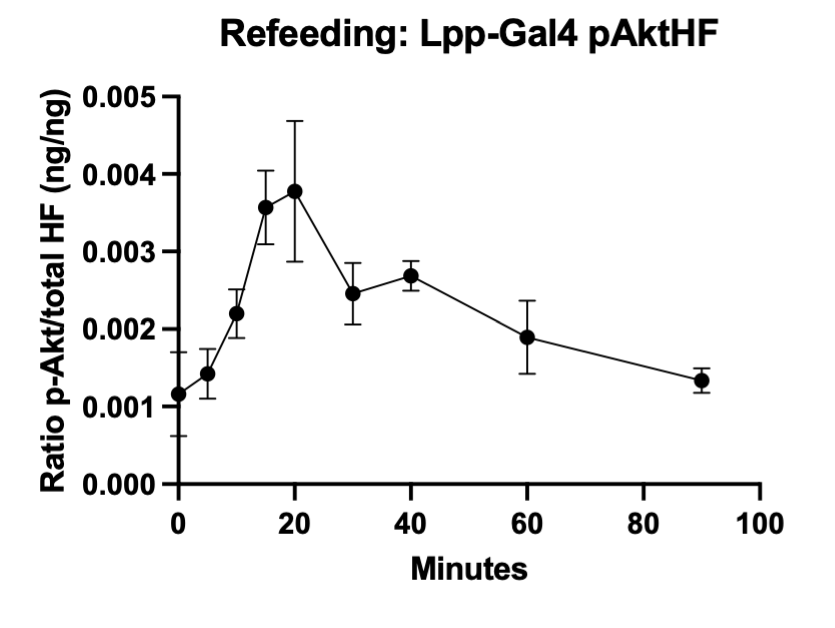

Supplement: S9 Fig — The genotype of virgin females used in these experiments is Ilp2-LexA, Tubp-Gal80TS/+; Lpp-Gal4, UAS-AktHF/UAS-w.RNAi (n = 4 flies per time point). (TIFF) [file pgen.1010619.s009.tiff]

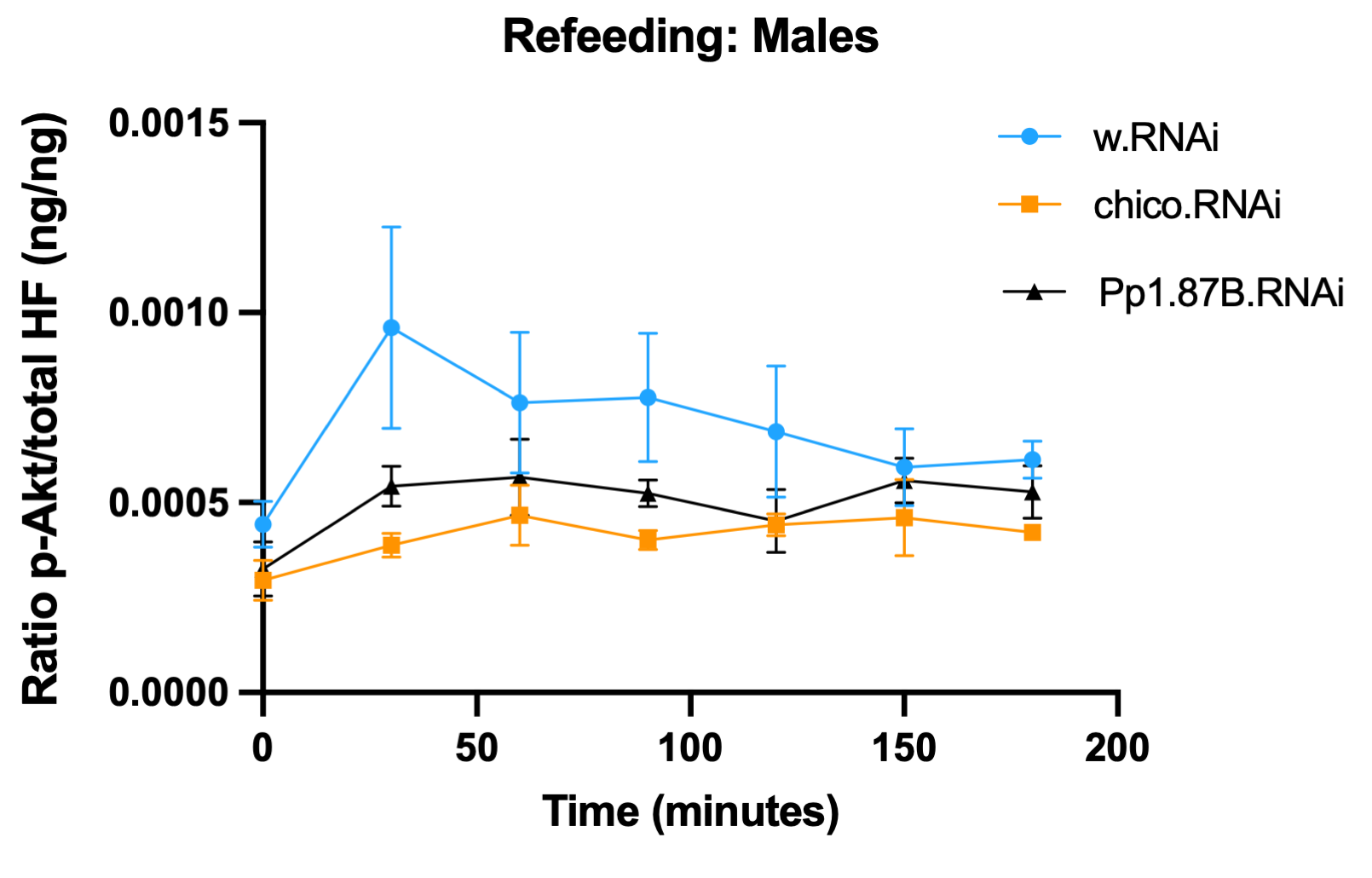

Supplement: S10 Fig — (TIFF) [file pgen.1010619.s010.tiff]

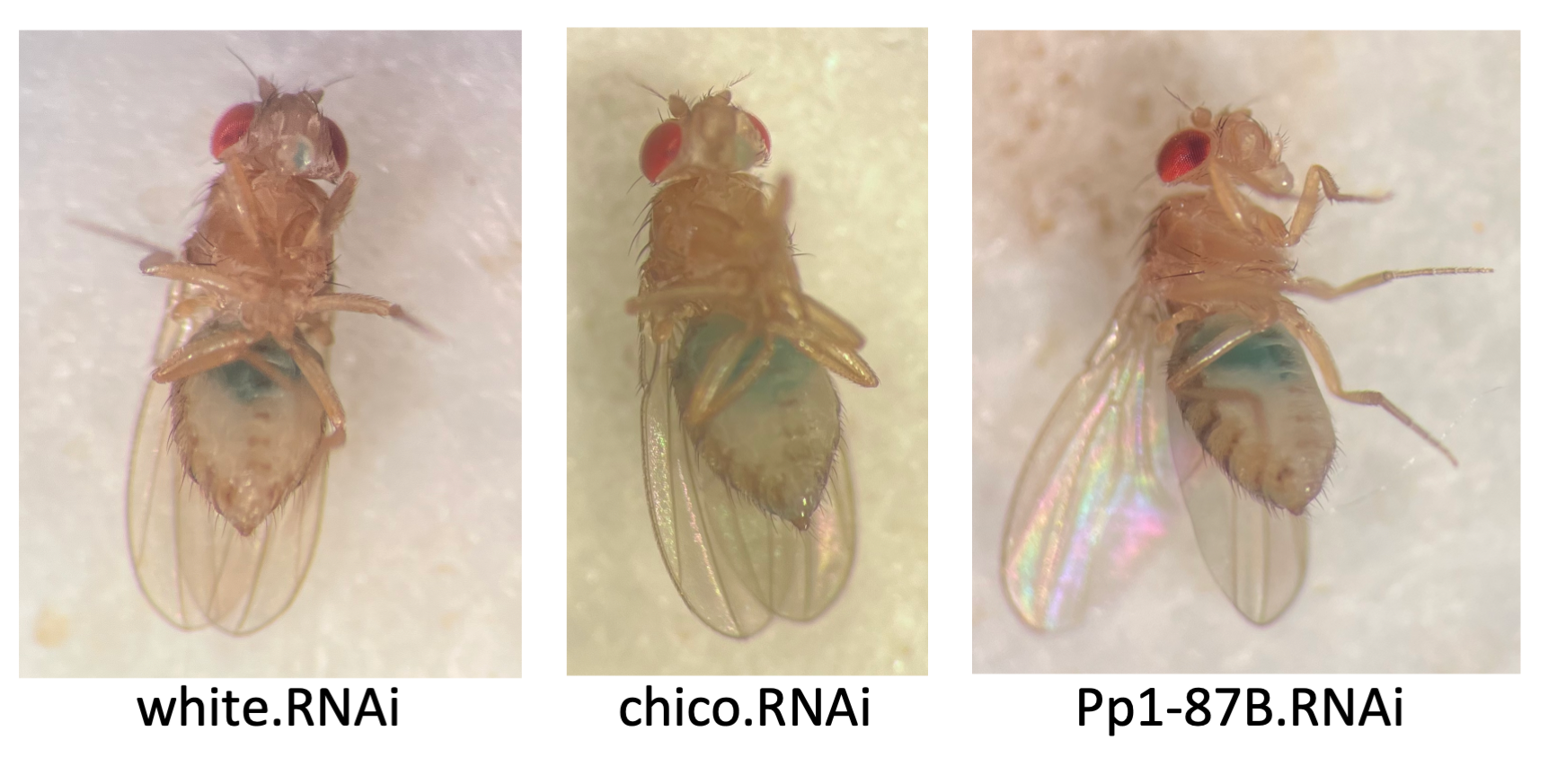

Supplement: S11 Fig — The ingested food can be visualized in the abdomen. Qualitatively, genotype differences did not affect the amount of food consumed. (TIFF) [file pgen.1010619.s011.tiff]
