# Supplementary material for: A genetic strategy to measure insulin signaling regulation and physiology in Drosophila
Source: PLoS Genet. 2023 Feb 2;19(2):e1010619. doi: 10.1371/journal.pgen.1010619 (PMC9928101; doi:10.1371/journal.pgen.1010619)
Supplement: S1 Methods — (DOCX) [file pgen.1010619.s012.docx]

**S1 Methods:**

**Dilutions and loading volumes for different drivers:**

Different tissue-specific drivers led to different levels of AktHF expression, so the volumes and dilutions of samples were adjusted to remain in the linear detection range of the assay (Fig 1C).

| **Driver** | | **Loading volume, total AktHF (µL)** | **Loading volume, pAktHF (µL)** |
| --- | --- | --- | --- |
| r5-LexA | In vivo | Diluted 1:200, loaded 10 | 20  20 |
|  | Ex vivo | Diluted 1:40, loaded 10 |  |
| Lpp-Gal4 | In vivo | Diluted 1:100, loaded 2.5 | 5  5 |
|  | Ex vivo | Diluted 1:50, loaded 2.5 |  |
| Mhc-LexA | In vivo | Diluted 1:100, loaded 20 | 20 |

**Quantification of protein, glucose, glycogen, and triglyceride content in single flies**

Virgin females with indicated genotypes were incubated at 30°C for 4 days, then incubated at 22°C for 24 hours on a fresh food-containing vial before the assay began.

A single fly was ground in 45 μl of ice-chilled PBS containing 1% Triton X-100 (PBTx1%) and centrifuged at the maximum speed for 30 seconds. 2 μl of the clear lysate was diluted in 40 μl of PBTx1%, and 20 μl of the diluted sample or BSA standards at 0 to 2,000 μg/ml were placed on 96 well assay plate. 100 μl of WR reagent (Thermo Scientific 23227) was added to each sample, and the assay plate was incubated at 37°C for 30 minutes. The absorbance at 562 nm was measured on the microplate reader. Total protein content per fly was calculated by μg/ml x 20 x 0.045 ml/fly. The rest of the undiluted clear lysate was transferred to PCR tube, and heat-inactivated at 70°C for >5 minutes immediately. The heat-denatured sample was mixed well by pipetting up and down several times.

To measure the triglyceride content, 20 μl of the heat-inactivated sample or Glycerol standards (Sigma-Aldrich G7793-5ML) at 0 to 2,500 μg/ml was mixed with 20 μl of Triglyceride Reagent (Sigma-Aldrich T2449-10ML) in a PCR tube, and incubated at 37°C for 30 minutes. The reaction was centrifuged at maximum speed for 30 seconds, and 20 μl of the clear sample or standards was placed on 96 well assay plate. 80 μl of Free Glycerol Reagent (Sigma-Aldrich F6428-40ML) was added to each sample, and the assay plate was incubated at 37°C for 10 minutes. The absorbance at 549 nm was measured on the microplate reader, and triglyceride content was calculated per fly by μg/ml x 0.045 ml/fly.

To measure the glucose content, the rest of the heat-inactivated sample was centrifuged at the maximum speed for 5 minutes. 5 μl of the clear sample, glucose standards at 0 to 4,000 μg/ml, or glycogen standards at 0 to 4,000 μg/ml was placed in a 96 well UV assay plate (Corning 3635). 200 μl of Glucose Hexokinase Reagent (Thermo Scientific TR15421) was added to each sample, and the assay plate was incubated at 37°C for 20 minutes. The absorbance at 340 nm was measured on the microplate reader, and the glucose content of each fly was calculated by μg/ml x 0.045 ml/fly. To measure glycogen content, 10 μl of water-diluted Amyloglucosidase (Sigma-Aldrich A7096) at (1:10,000) was added to each sample and standard. The plate was sealed and incubated at 37°C for 16 hours, and the absorbance at 340 nm was measured on the microplate reader. The combined glucose and glycogen content per fly was calculated by μg/ml x 0.045 ml/fly, and the glycogen content per fly was determined by subtracting the glucose content per fly measured in the prior assay.

**Adult eclosion timing assay**

To compare the developmental timing of flies harboring different transgenes, 5 virgin females of *yw* were mated to 5 homozygous males of the following genotypes independently for 5 days at 18°C: (1) *yw ; LexAop-AktHF*, (2) *yw ; r5-LexA, Tubp-Gal80^TS^ ; Ilp2-Gal4, LexAop-AktHF,* (3) *yw ; UAS-AktHF.* Eggs laid from each intercross were collected for 24 hours at 18°C, then further incubated at 18°C for 24 days with 12 hour-light and 12 hour-dark cycles. Eclosed adult flies were counted daily at 3 hours after the start of the last 12 hour-light cycle.

**Starvation survival assay**

To compare the starvation resistance of flies harboring different transgenes, virgin females with indicated genotypes were incubated at 30°C for 4 days, then incubated at 22°C for 24 hours on a fresh food-containing vial before the start of the assay. 5 day-old conditioned females were placed on a vial containing 5 ml of 2% agar and 0.1% Methylparaben, and incubated at 22°C during the starvation for 144 hours under 12 hour-light and 12 hour-dark cycles. The number of the dead flies were counted twice daily at 3 hours after the start of the last 12 hour-light or dark cycle.

**Western Blotting**

F1 male flies of *r5-LexA, Tubp-Gal80^TS^ ; Ilp2-Gal4, LexAop-AktHF* x *UAS-CD4-tdGFP* were fed at 30°C for 5 days and fasted at 22°C for 24 hours. After random sampling of 10 fasted flies without CO_2_ exposure, the rest of the fasted flies were on 1 M glucose with blue food color for 10 minutes, then re-fasted for 20 minutes. 1, 2, 3, or 4 fasted or refed flies were ground in ice-cold 100 µl PBS with 1% Triton-X-100, 100mM sodium fluoride, and 5mM sodium orthovanadate. After centrifuging at 21,000g for 30 seconds at 4^o^C, the supernatant was used for the ELISA and western blotting. 5 μl of the supernatant or protein standards (Bio-Rad 1610377) was separated by SDS-PAGE (Any kD Mini-PROTEAN, Bio-Rad 4569033), and transferred to the PVDF membrane. The membrane was sequentially probed for anti-HA-HRP (1:50,000), anti-phospho-Akt.S505 (1:5,000)/anti-rabbit-HRP (1:20,000), and anti-Actin-HRP (1:5,000) to detect HRP signal on X-ray films by chemiluminescent substrate (Thermo Fisher 34577). 5 μl of the same supernatant were used for phospho-AktHF ELISA, and 10 μl of (1:100) diluted supernatant were used for total AktHF ELISA with two technical duplicates for each ELISA assay.
